# Supplementary material for: Bayesian machine learning enables discovery of risk factors for hepatosplenic multimorbidity related to schistosomiasis
Source: Nat Commun. 2026 Mar 3;17:3377. doi: 10.1038/s41467-026-69528-4 (PMC13066555; doi:10.1038/s41467-026-69528-4)
Supplement: Supplementary file 1 — Supplementary Information [file 41467_2026_69528_MOESM1_ESM.pdf]

Supplementary

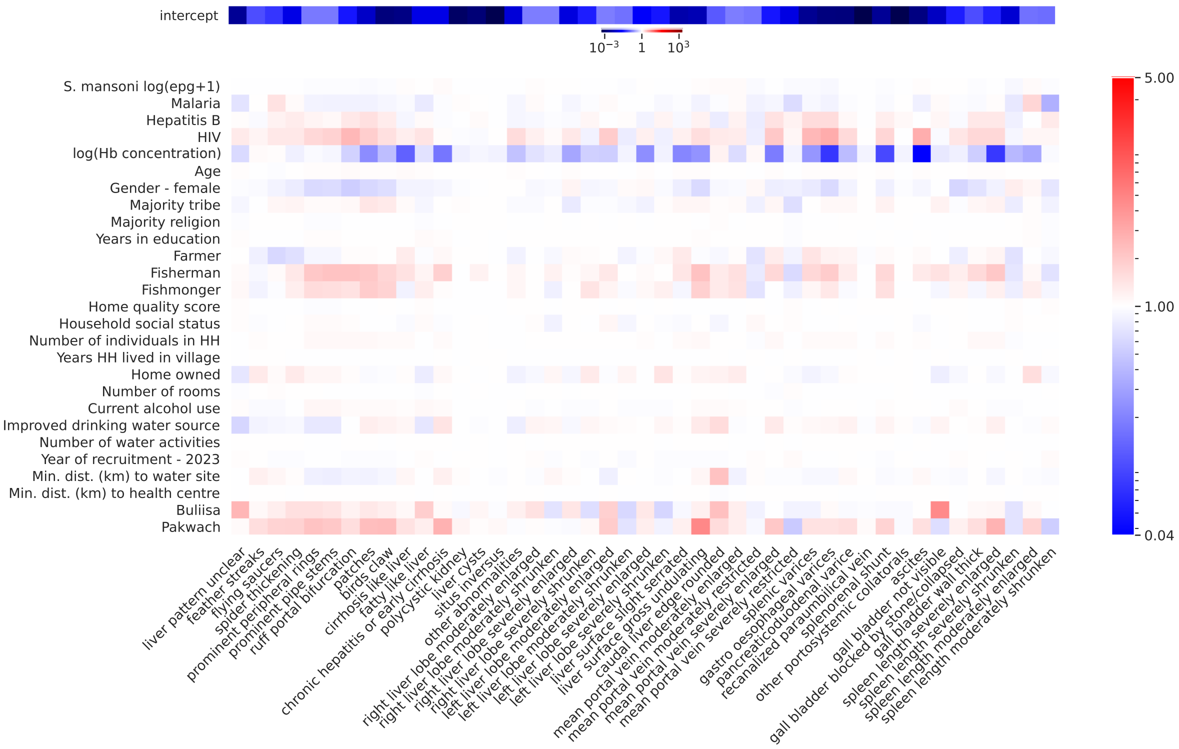

Figure S1: Mean posterior odds ratios of m_{ij}/σ_j.
Each column shows to the mean odds ratios of the covariates for predicting the corresponding condition of that column. Results are computed over the full dataset.

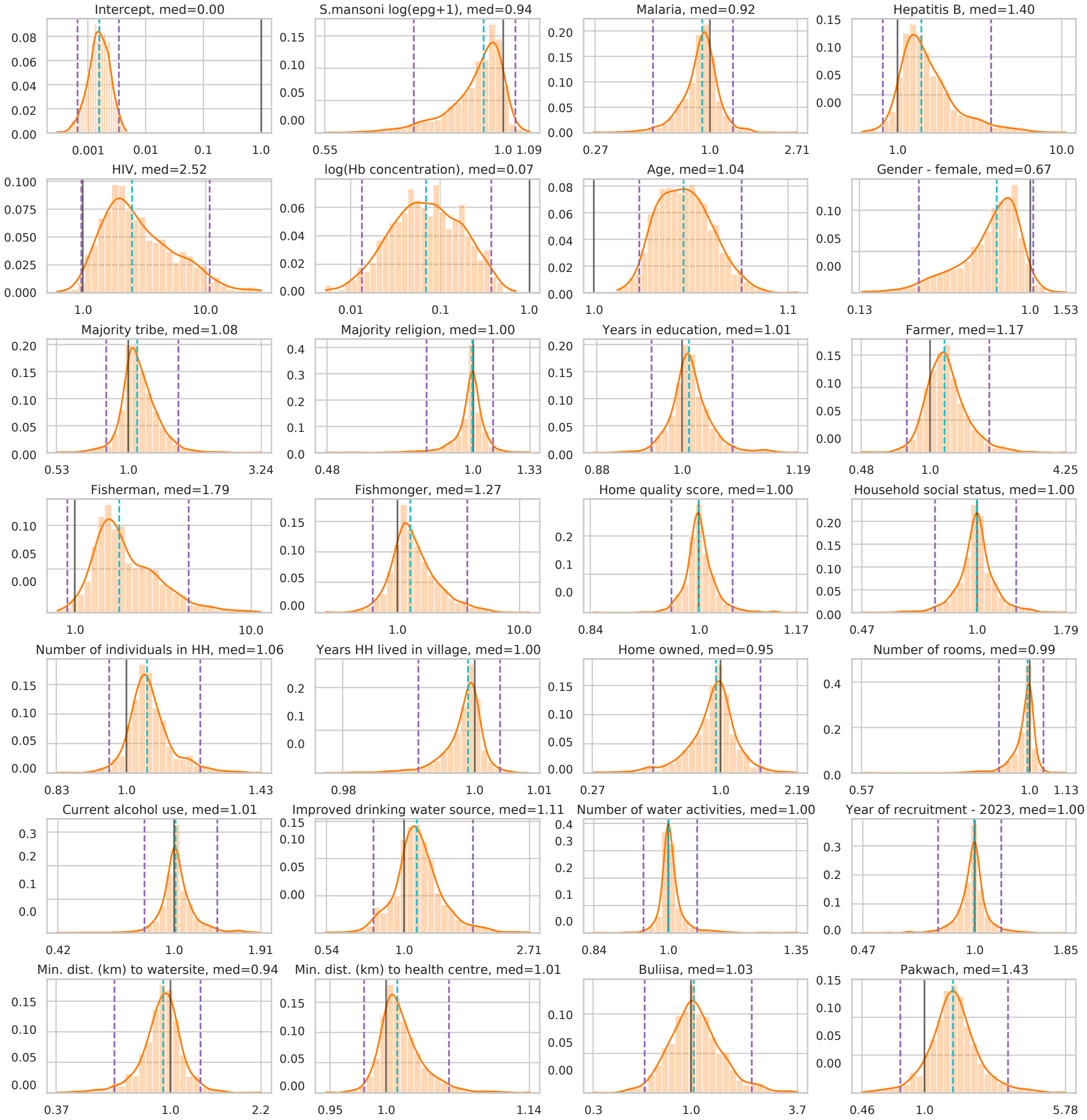

Figure S2: Posterior distribution of multitask regression odds ratios for gastro-oesophageal varices.

The distributions correspond to the column of regression coefficients for gastro-oesophageal varices in Fig. S1. Black lines mark odds ratio 1, cyan dashed lines mark the medians, and purple dashed lines mark the 95% highest posterior density credible intervals. Results are computed over the full dataset.

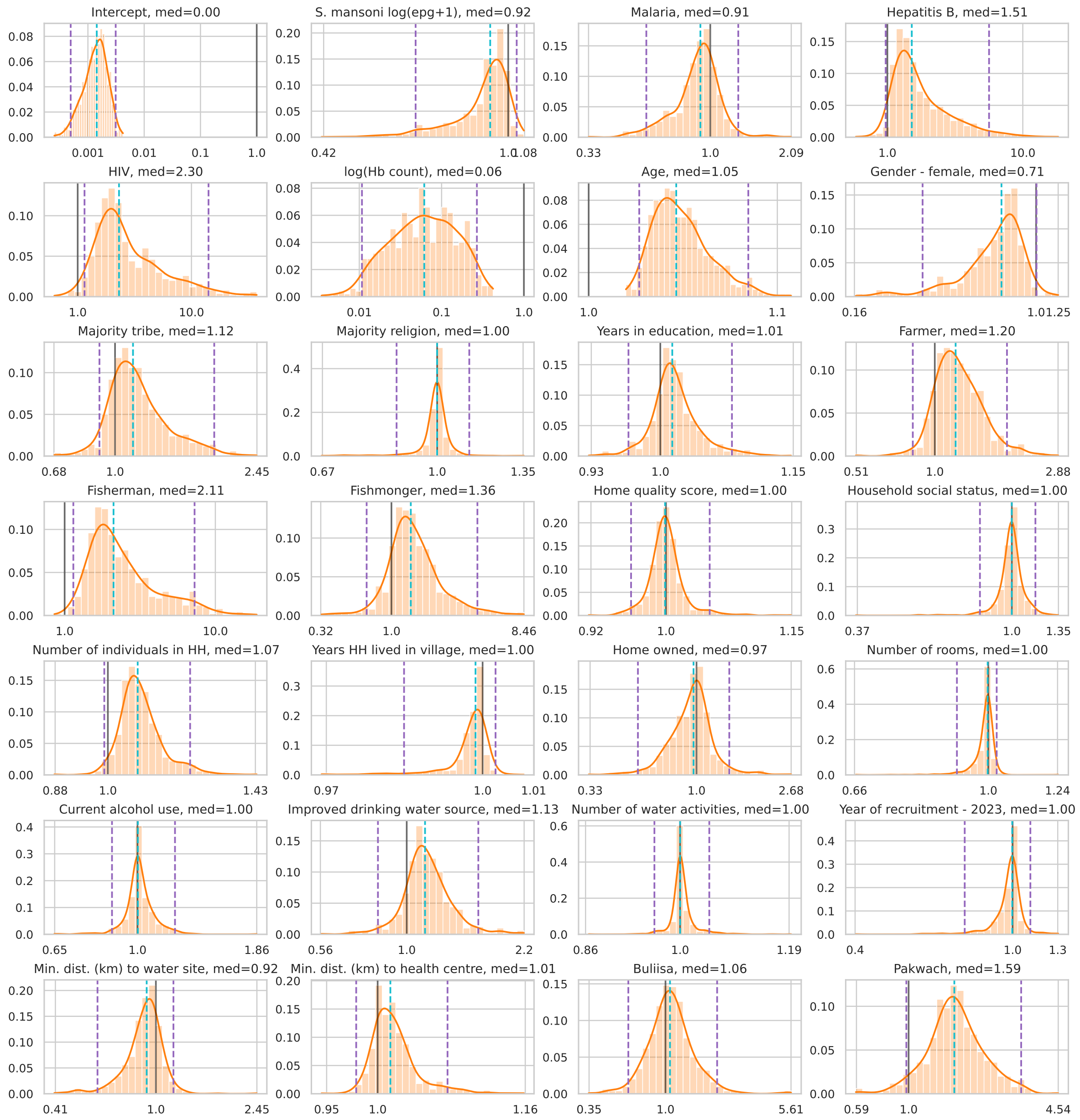

Figure S3: Posterior distribution of multitask regression odds ratios for gastro-oesophageal varices using alternative priors.

Priors assigned were Beta(0.5,0.5), t_3 distribution with non-fixed scale, and $\Gamma(1, 3)$ for the inclusion probabilities, regression weights, and graph convolution parameter respectively (Fig. S2 used default priors specified in Methods). Black lines mark odds ratio 1, cyan dashed lines mark the medians, and purple dashed lines mark the 95% highest posterior density credible intervals. Results are computed over the full dataset.

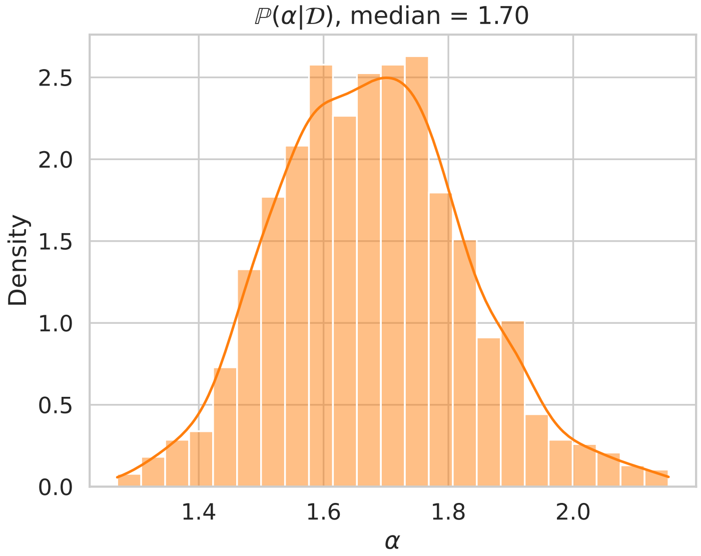

Figure S4: Posterior distribution of α from multitask model. Results are computed over the full dataset.

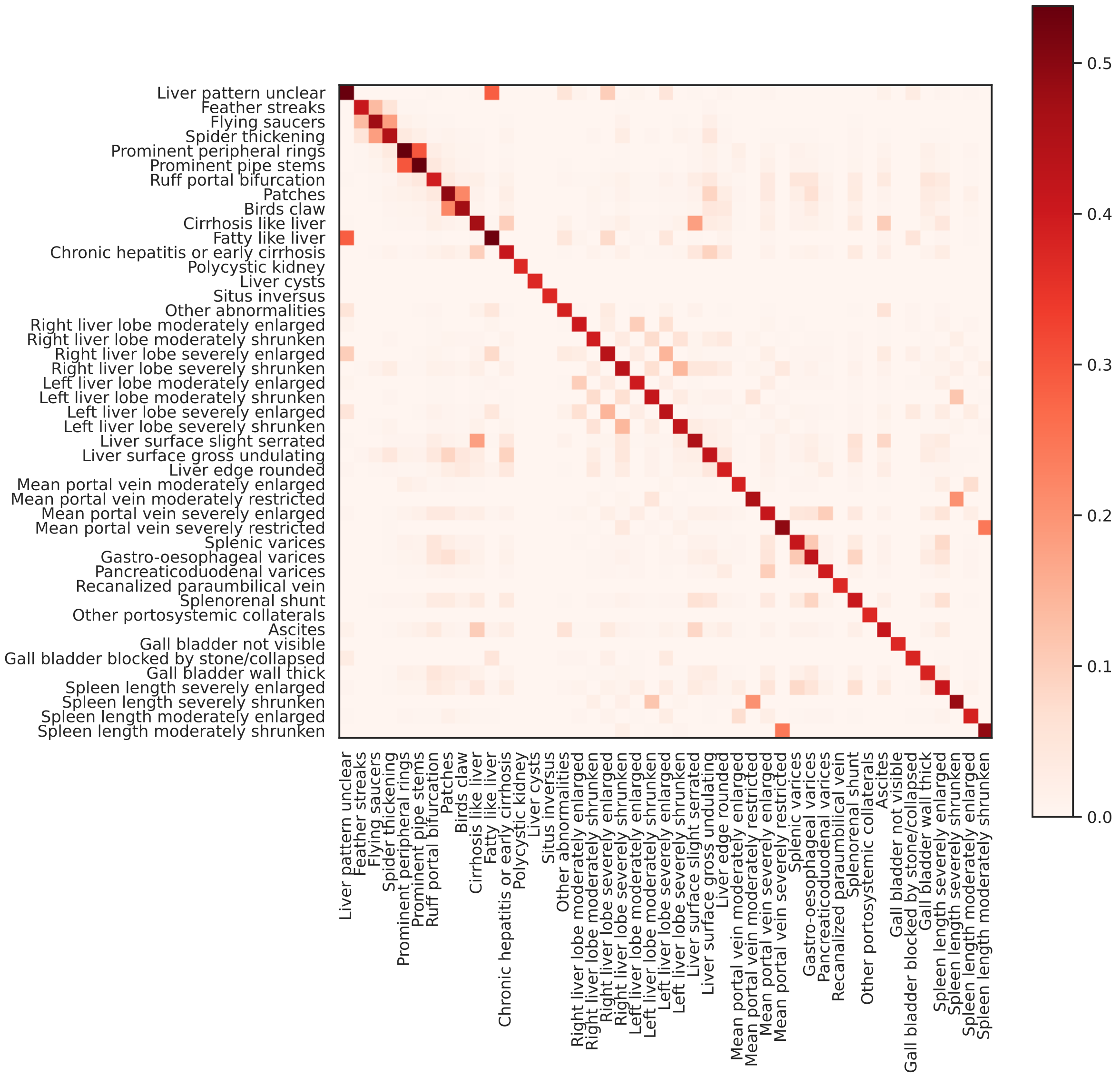

Figure S5: Relative probabilities between all conditions.
Probabilities are determined by the graph convolution matrix $f_\alpha(\mathcal{G})$ using median α - see Fig. S4. Diagonal indicates self influence. Results are computed over the full dataset.

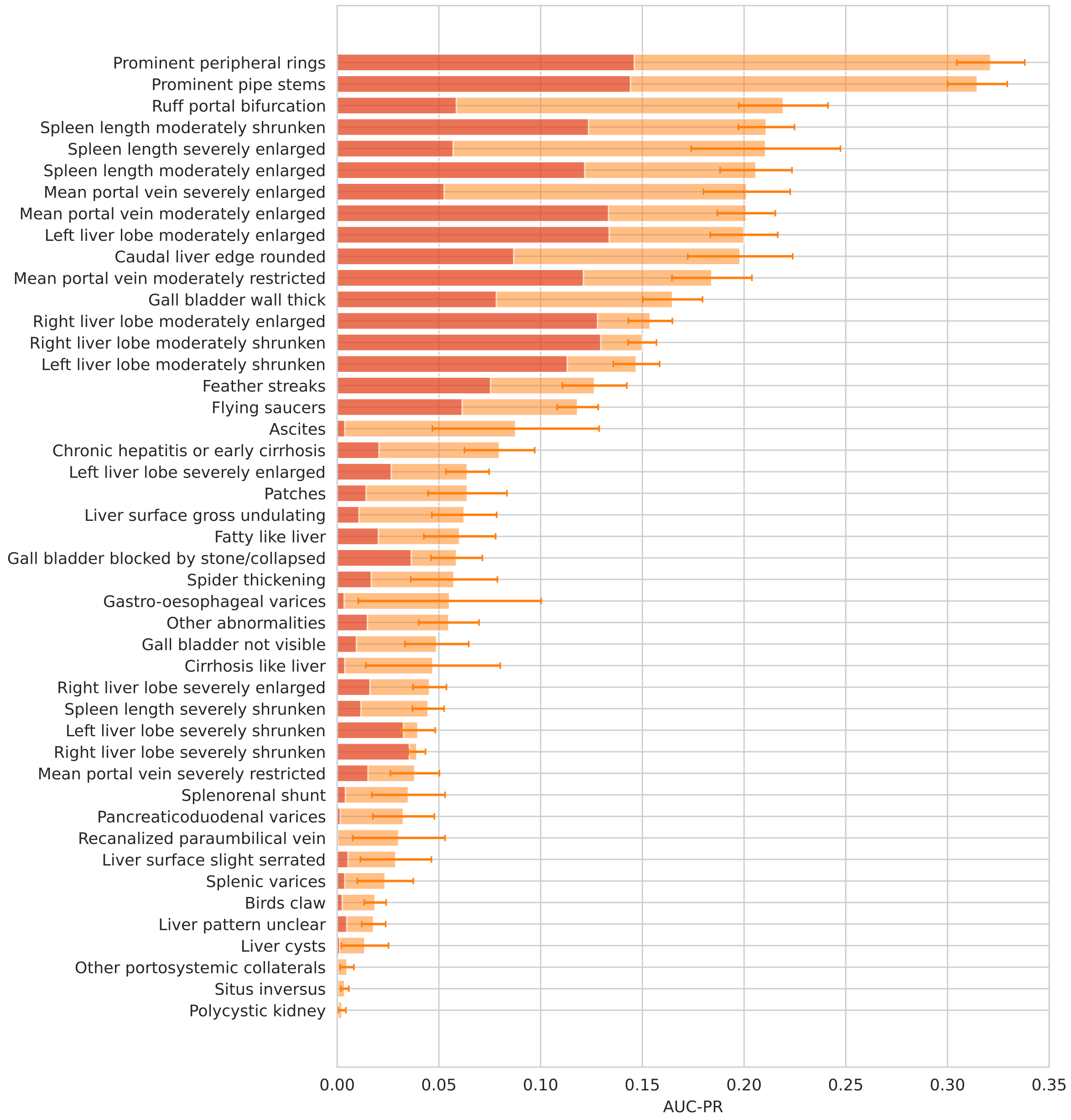

Figure S6: AUC-PR of Bayesian multitask model against prevalence of conditions. Orange: AUC-PR from the model prediction, Red: prevalence of the conditions. The ratio of AUC-PR and prevalence can be found in Fig. 3.

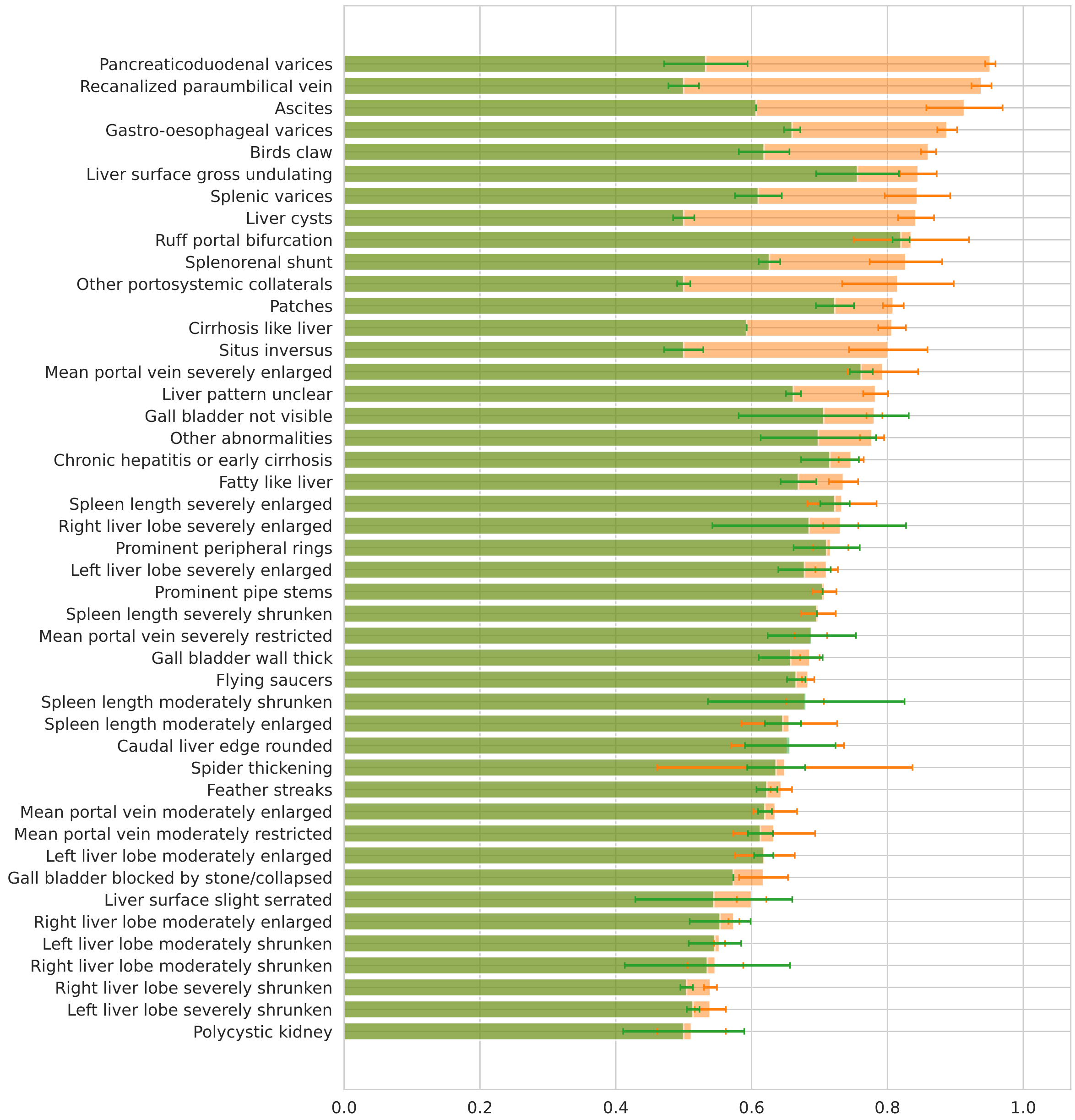

Figure S7: Conditions prediction AUCs of our Bayesian multitask learning (orange) and 45 separate logistic regression (green).
The AUC of each condition is averaged over 10 random training-testing splits. Average test AUC from multitask model (orange): 0.721 ± 0.011, from 45 single-output models (green): 0.636 ± 0.007. All AUCs reported were evaluated on 10 randomly sampled test sets of 50% of the data using models trained on the other 50%.

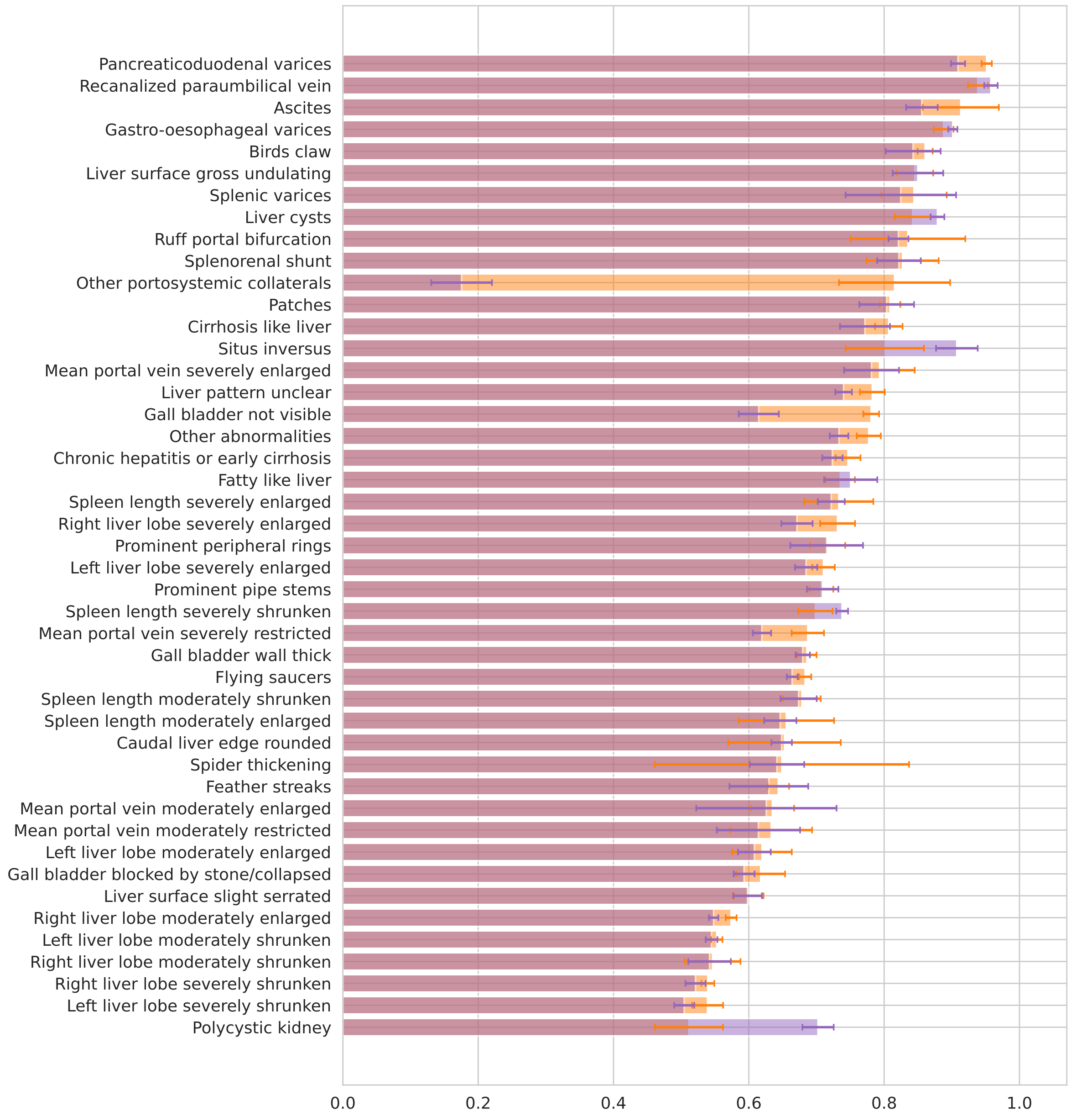

Figure S8: Conditions prediction AUCs of our Bayesian multitask learning (orange) and multitask neural network (purple).
The AUC of each condition is averaged over 10 random training-testing splits. Average test AUC from Bayesian multitask model (orange): 0.721 ± 0.011, from multitask neural network (green): 0.703 ± 0.012. All AUCs reported were evaluated on 10 randomly sampled test sets of 50% of the data using models trained on the other 50%.

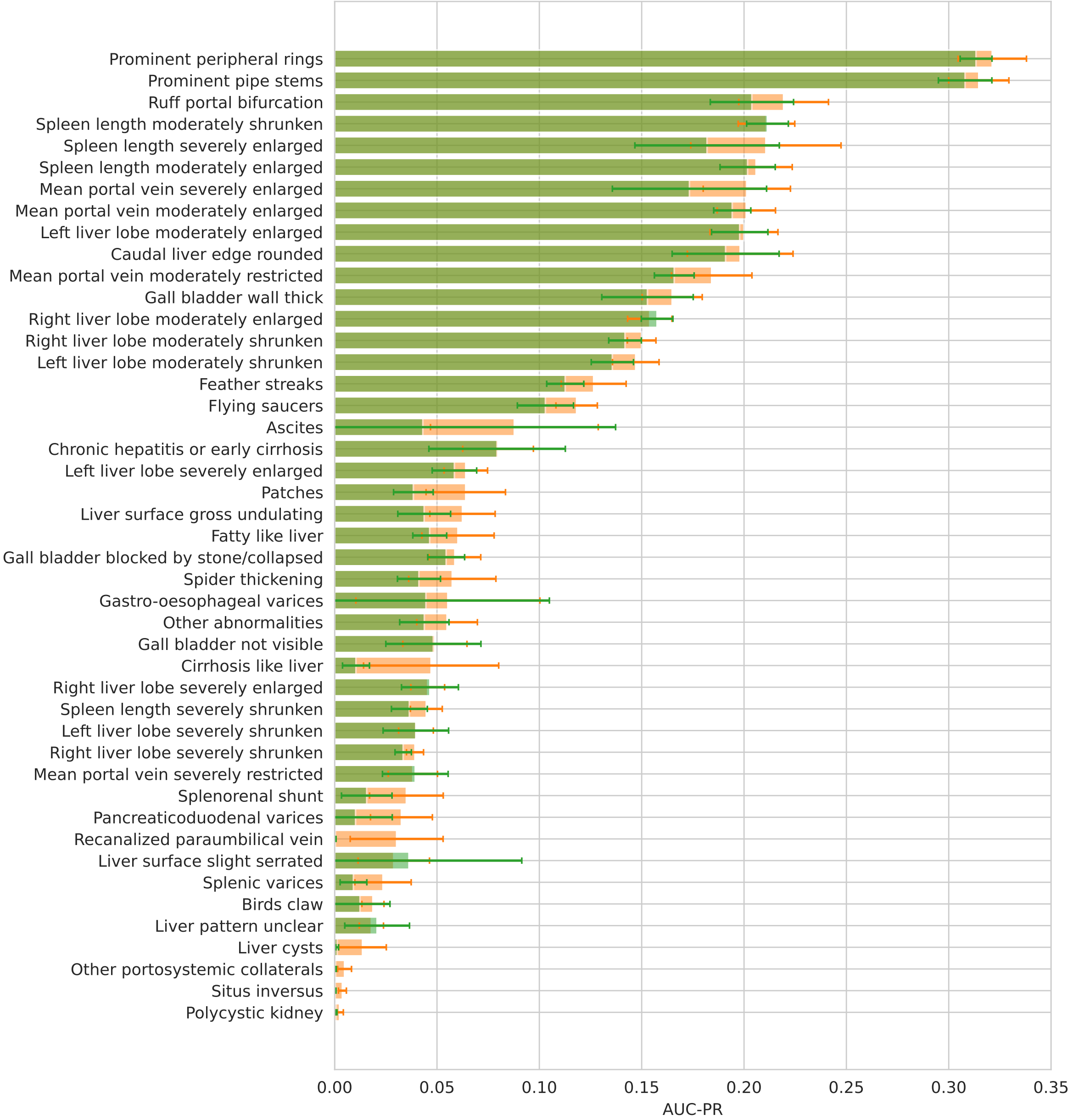

Figure S9: Conditions prediction AUC-PRs of our Bayesian multitask learning (orange) and 45 separate logistic regression (green).
The AUC-PR of each condition is averaged over 10 random training-testing splits. Average test AUC-PR from multitask model (orange): 0.103 ± 0.004, from 45 single-output models (green): 0.089 ± 0.003. All AUC-PRs reported were evaluated on 10 randomly sampled test sets of 50% of the data using models trained on the other 50%.

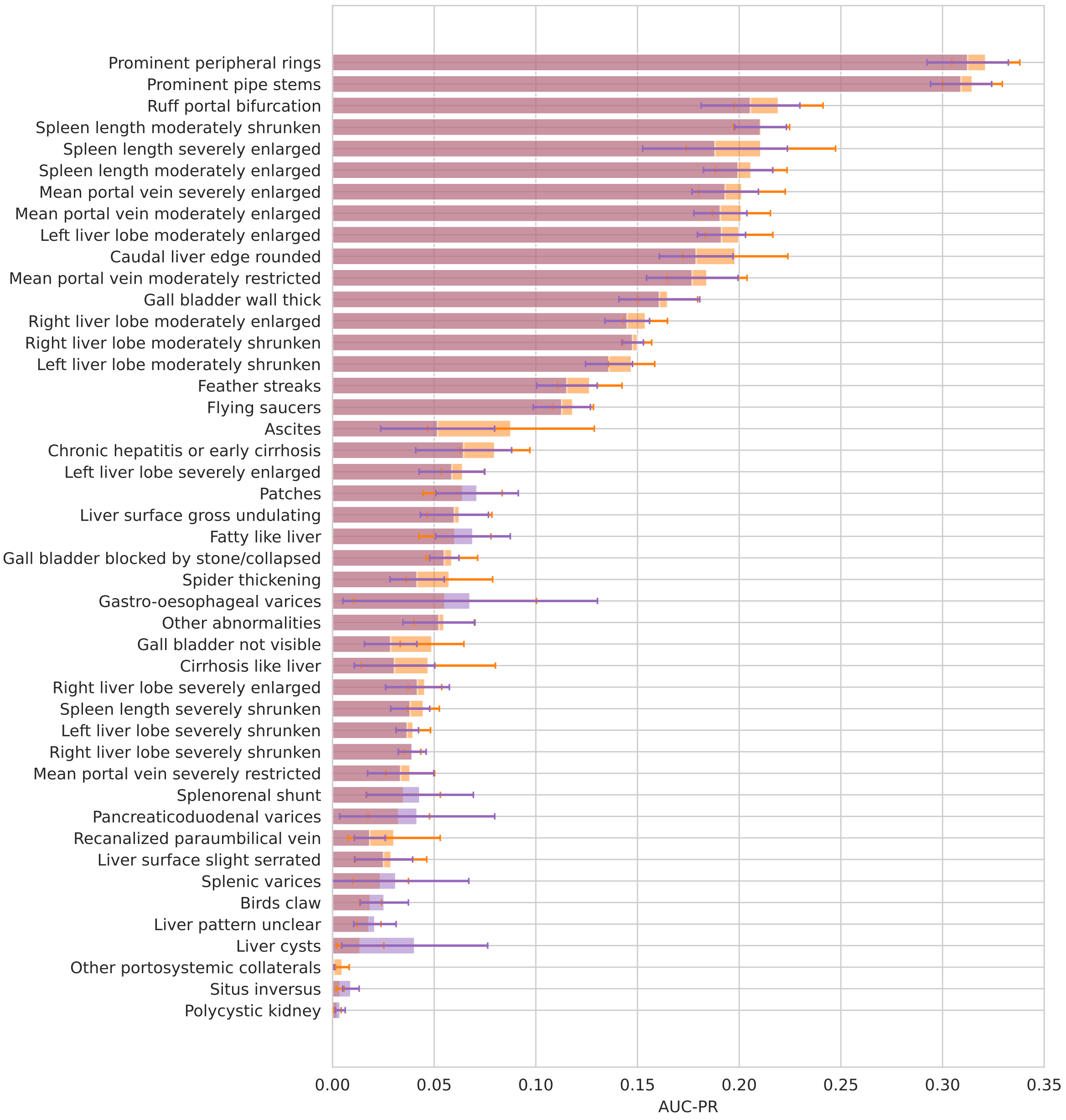

Figure S10: Conditions prediction AUC-PRs of our Bayesian multitask learning (orange) and multitask neural network (purple).
The AUC-PR of each condition is averaged over 10 random training-testing splits. Average test AUC-PR from Bayesian multitask model (orange): 0.103 ± 0.004, from multitask neural network (green): 0.099 ± 0.004. All AUC-PRs reported were evaluated on 10 randomly sampled test sets of 50% of the data using models trained on the other 50%.

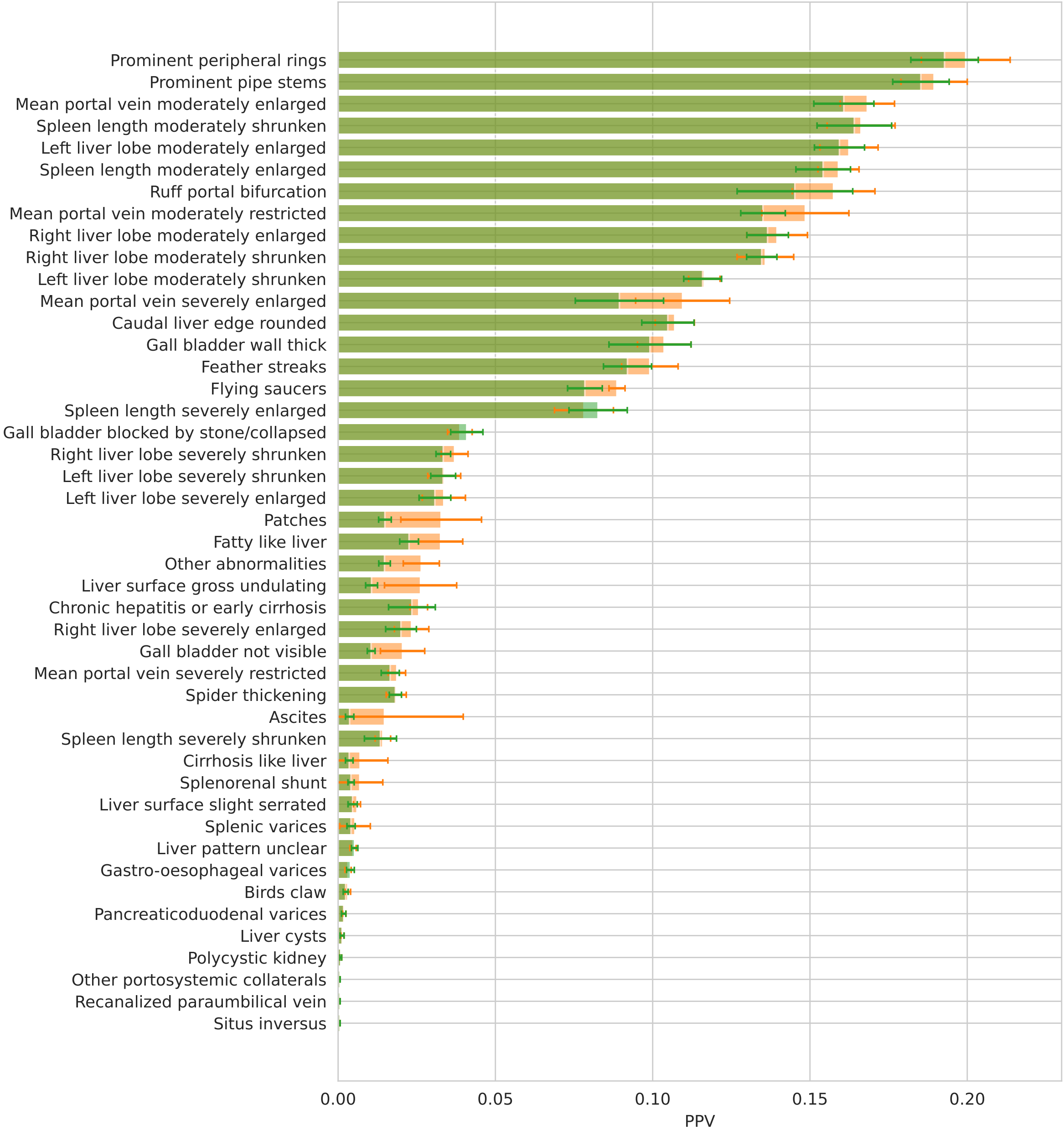

Figure S11: Conditions prediction PPVs of our Bayesian multitask learning (orange) and 45 separate logistic regression (green).
The PPV of each condition is averaged over 10 random training-testing splits. Average test PPV from multitask model (orange): 0.122 ± 0.003, from 45 single-output models (green): 0.103 ± 0.002. All PPVs reported were evaluated on 10 randomly sampled test sets of 50% of the data using models trained on the other 50%.

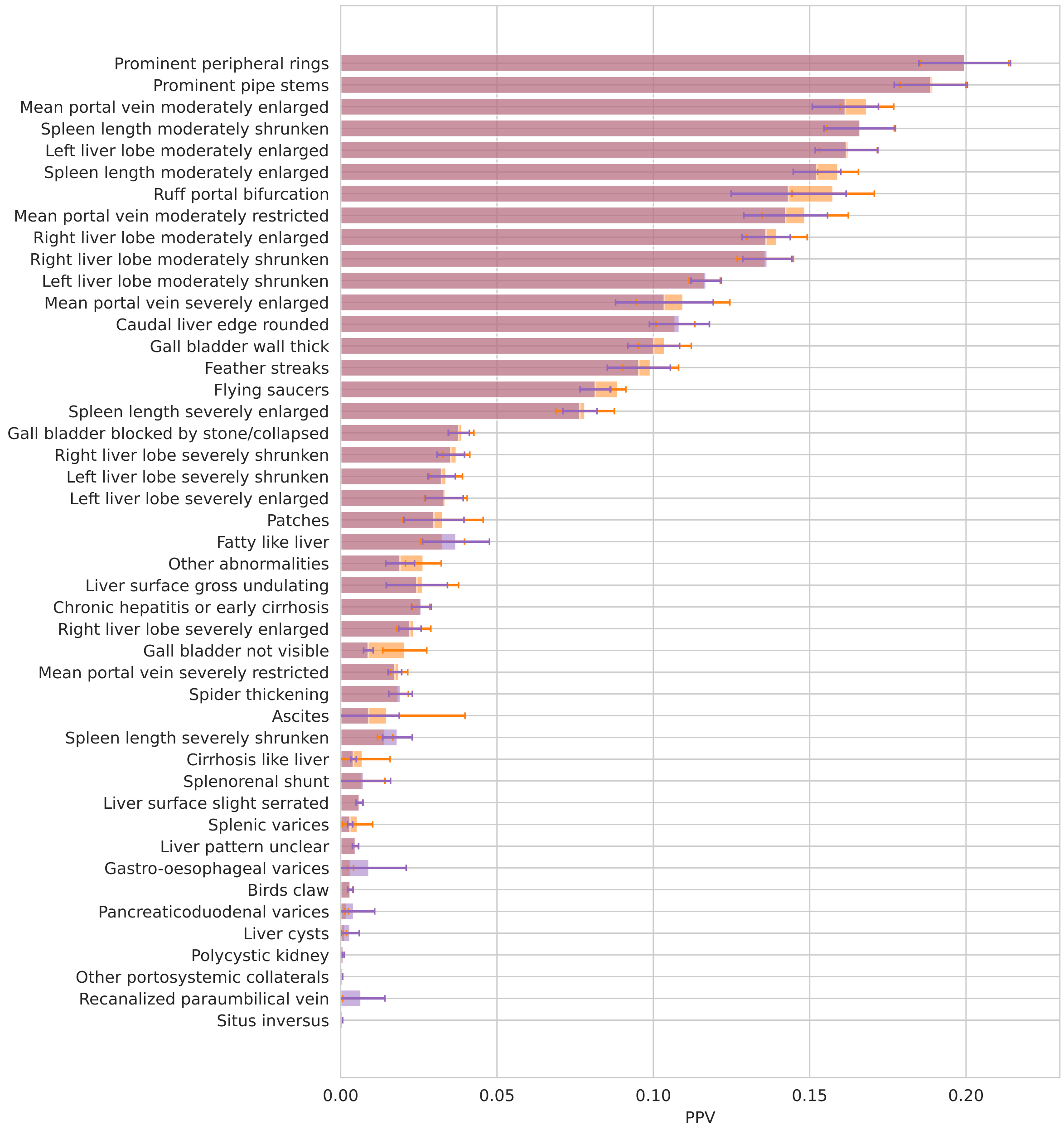

Figure S12: Conditions prediction PPVs of our Bayesian multitask learning (orange) and multitask neural network (purple).
The PPV of each condition is averaged over 10 random training-testing splits. Average test PPV from Bayesian multitask model (orange): 0.122 ± 0.003, from multitask neural network (green): 0.115 ± 0.003. All PPVs reported were evaluated on 10 randomly sampled test sets of 50% of the data using models trained on the other 50%.

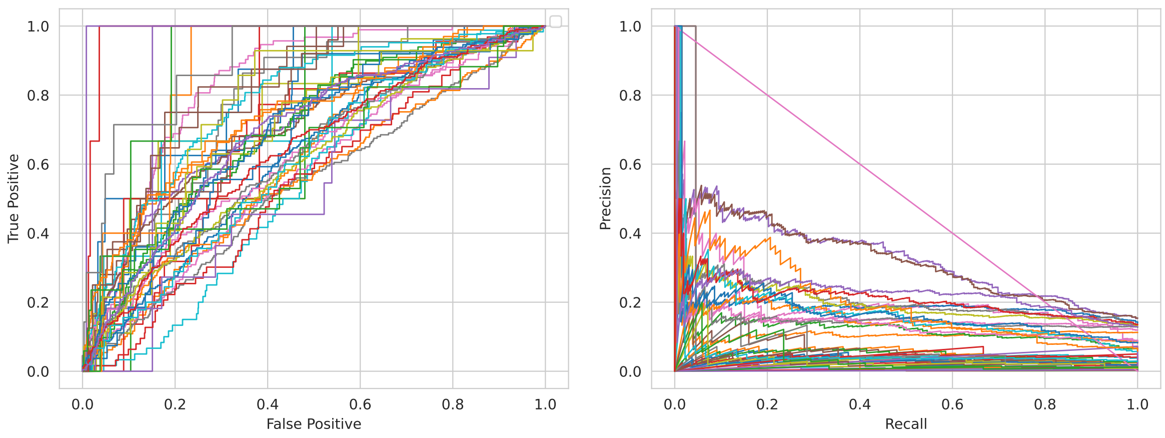

(a) (b)

Figure S13: AUC and AUC-PR curves of all 45 conditions.
(a) AUC curves, (b) AUC-PR curves, each colour represents a condition. The curves represent one of the 10 test splits.

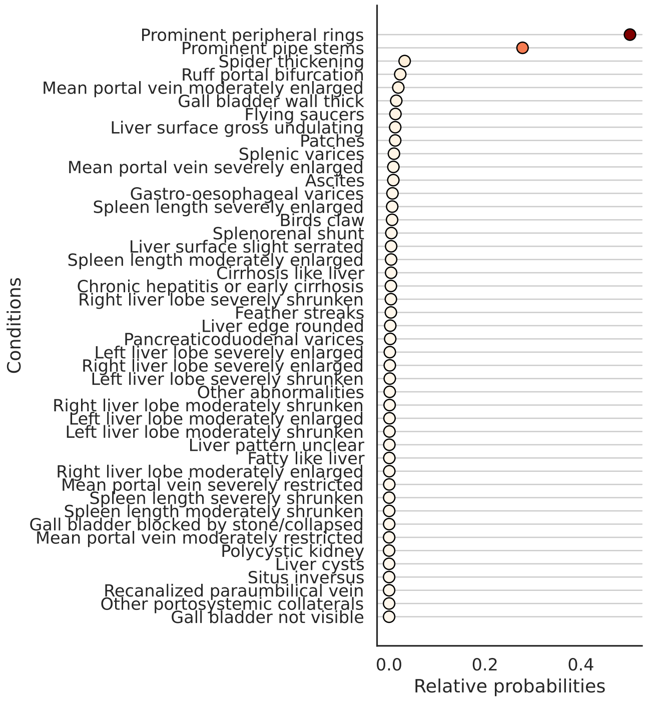

Figure S14: Influence probabilities for prominent peripheral rings liver pattern, grade C1.
Results are computed over the full dataset.

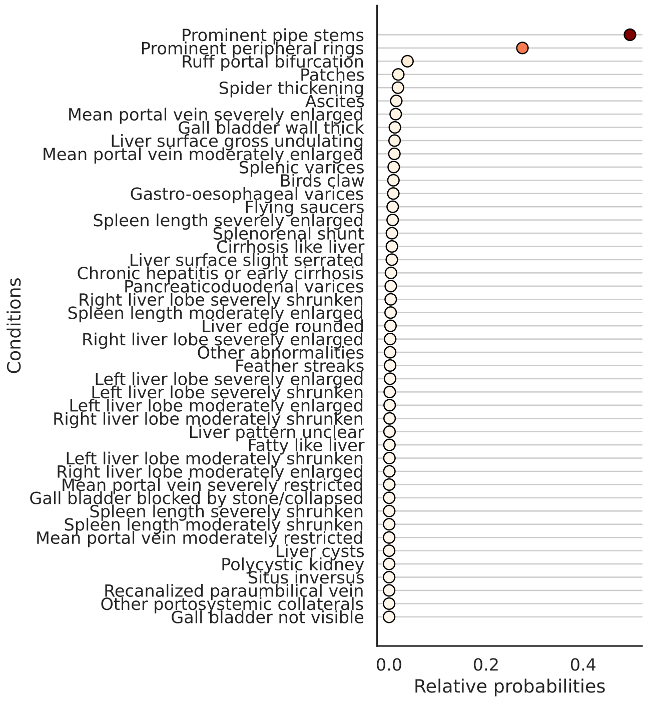

Figure S15: Influence probabilities for prominent pipe stems liver pattern, grade C2. Results are computed over the full dataset.

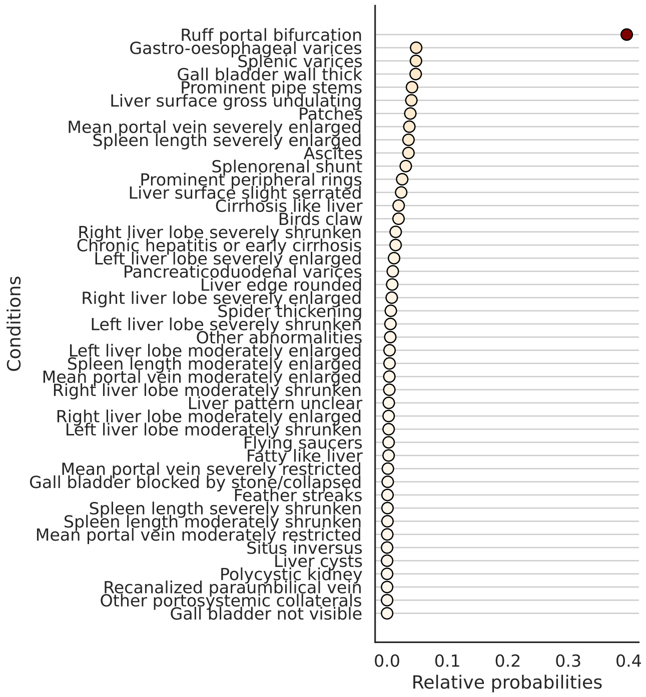

Figure S16: Influence probabilities for ruff portal bifurcation liver pattern, grade D. Results are computed over the full dataset.

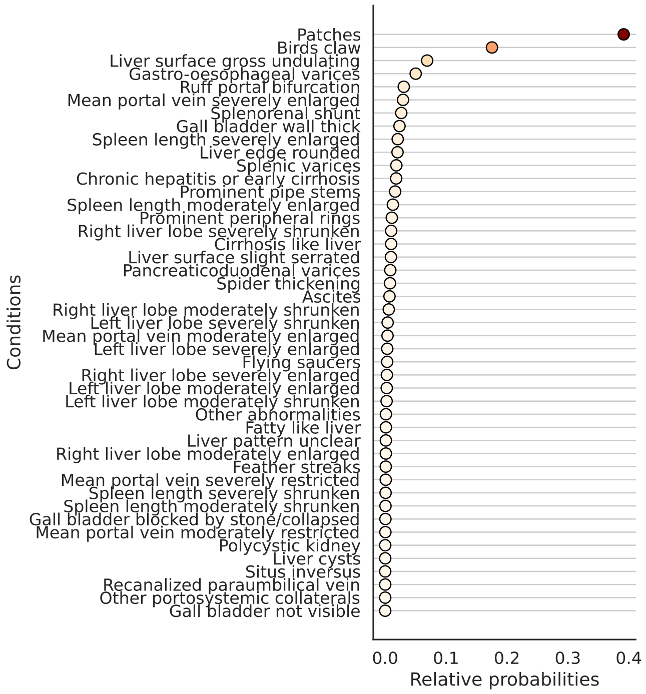

Figure S17: Influence probabilities for patches liver pattern, grade E. Results are computed over the full dataset.

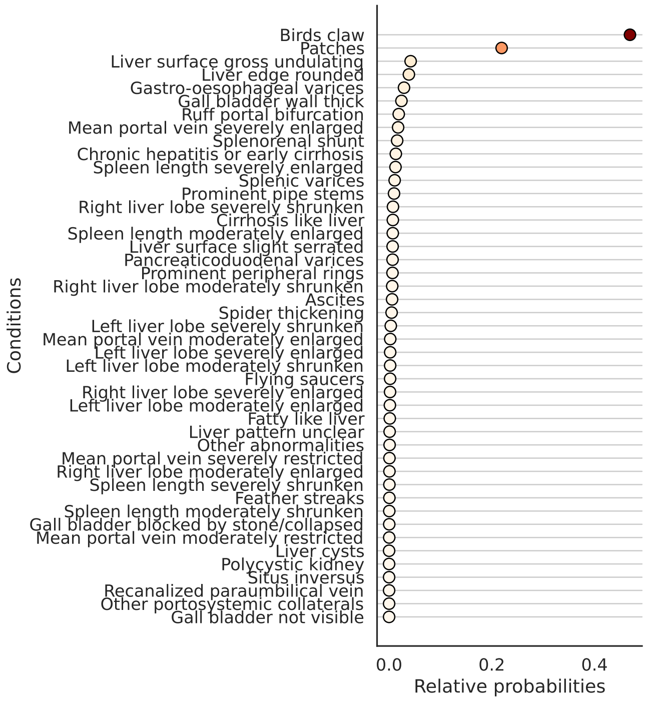

Figure S18: Influence probabilities for birds claw liver pattern, grade F. Results are computed over the full dataset.

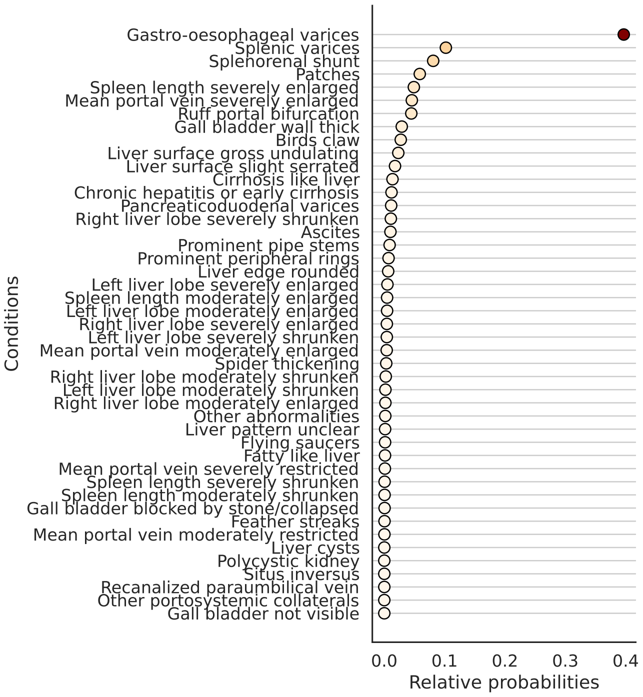

Figure S19: Influence probabilities for gastro-oesophageal varices. Results are computed over the full dataset.

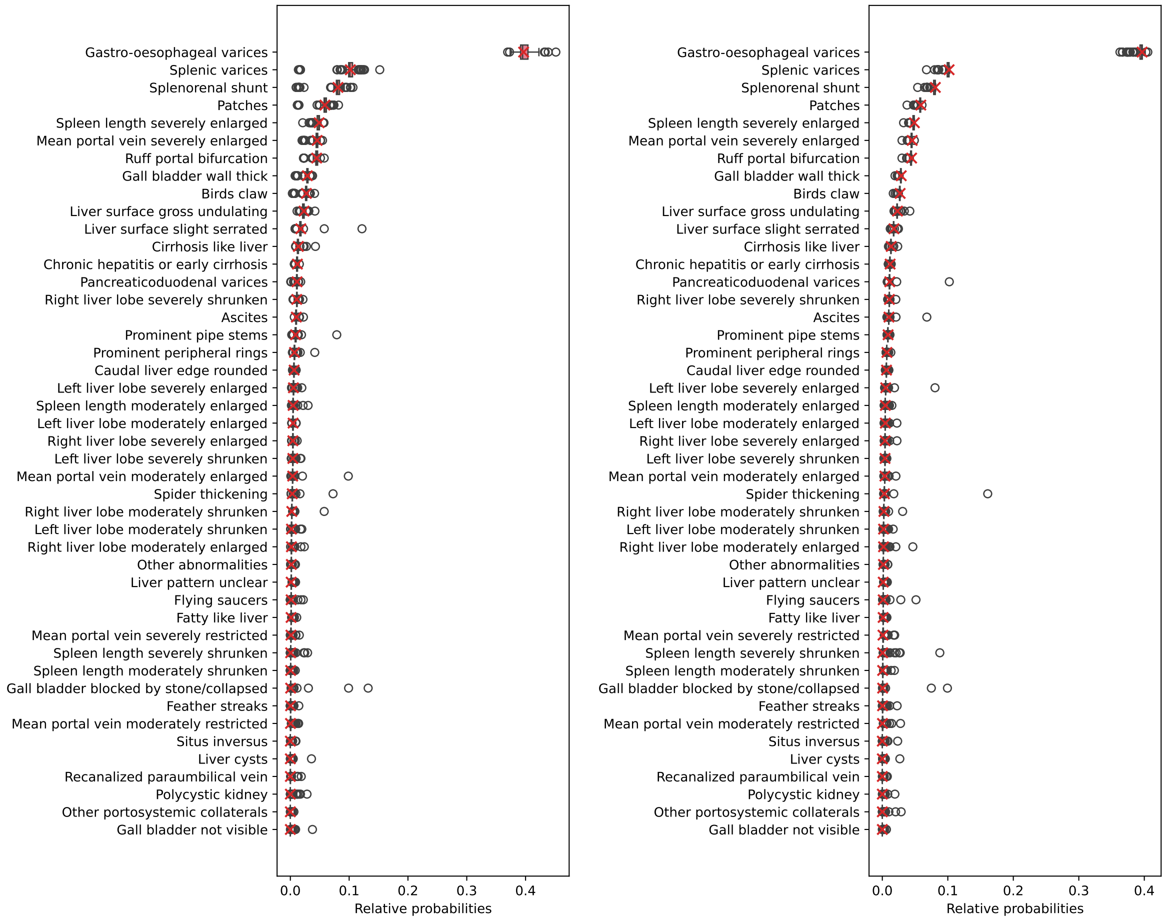

(a)

(b)

Figure S20: Influence probabilities for gastro-oesophageal varices under graph perturbation through random adding and removing 5% of edges.
(a) 5% edge addition and removal, (b) 5% edge addition, red X mark the original influence probabilities with no perturbation.

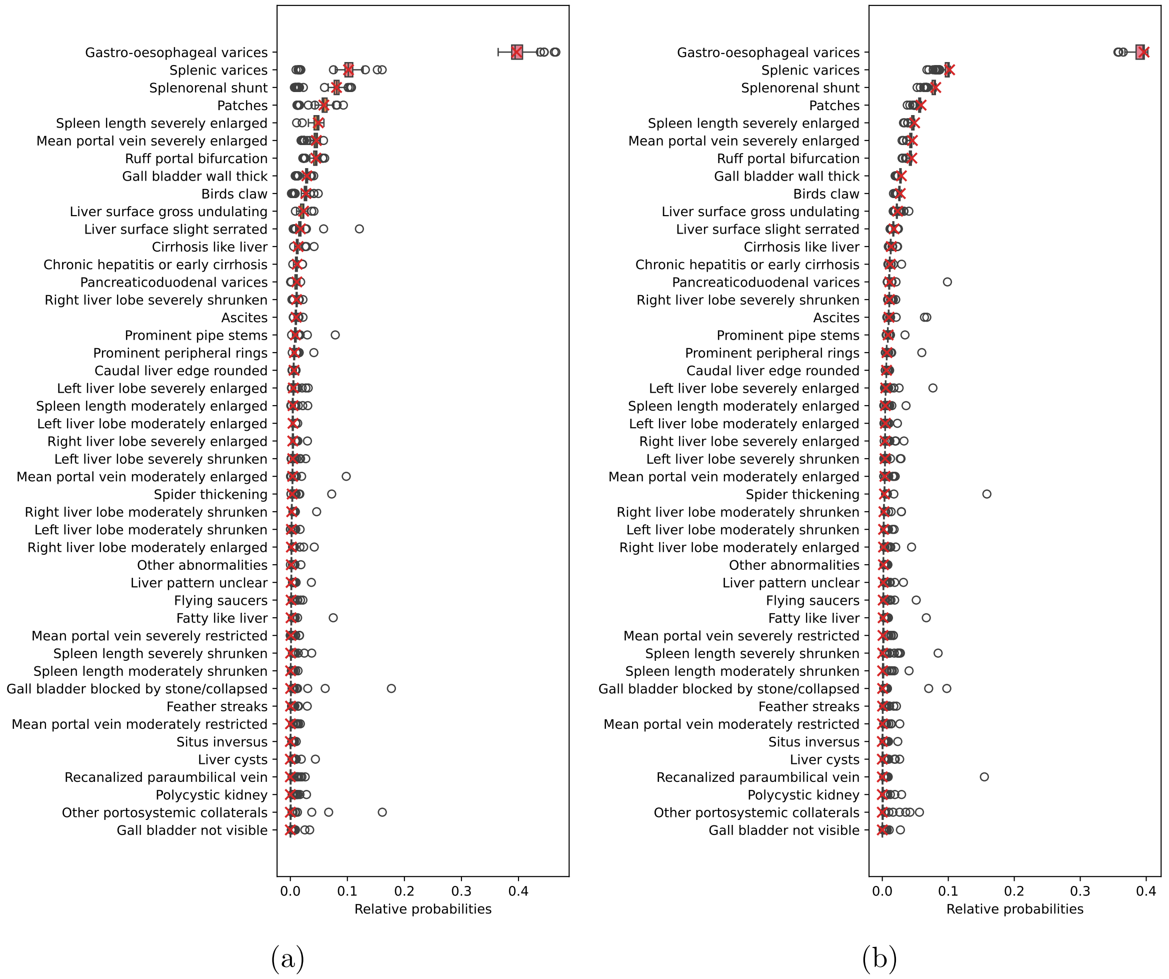

Figure S21: Influence probabilities for gastro-oesophageal varices under graph perturbation through random adding and removing 10% of edges.
(a) 10% edge addition and removal, (b) 10% edge addition, red X mark the original influence probabilities with no perturbation.

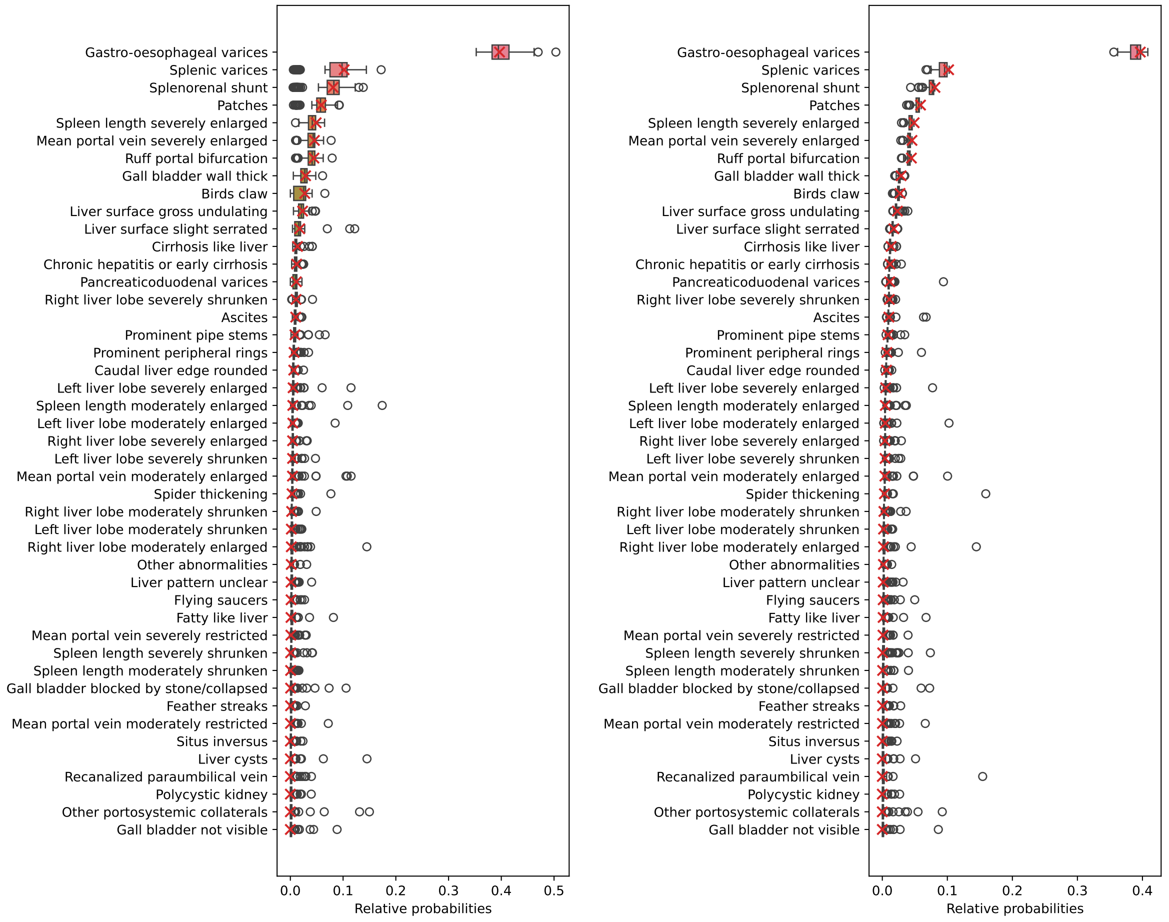

(a) (b)

Figure S22: Influence probabilities for gastro-oesophageal varices under graph perturbation through random adding and removing 20% of edges.
(a) 20% edge addition and removal, (b) 20% edge addition, red X mark the original influence probabilities with no perturbation.

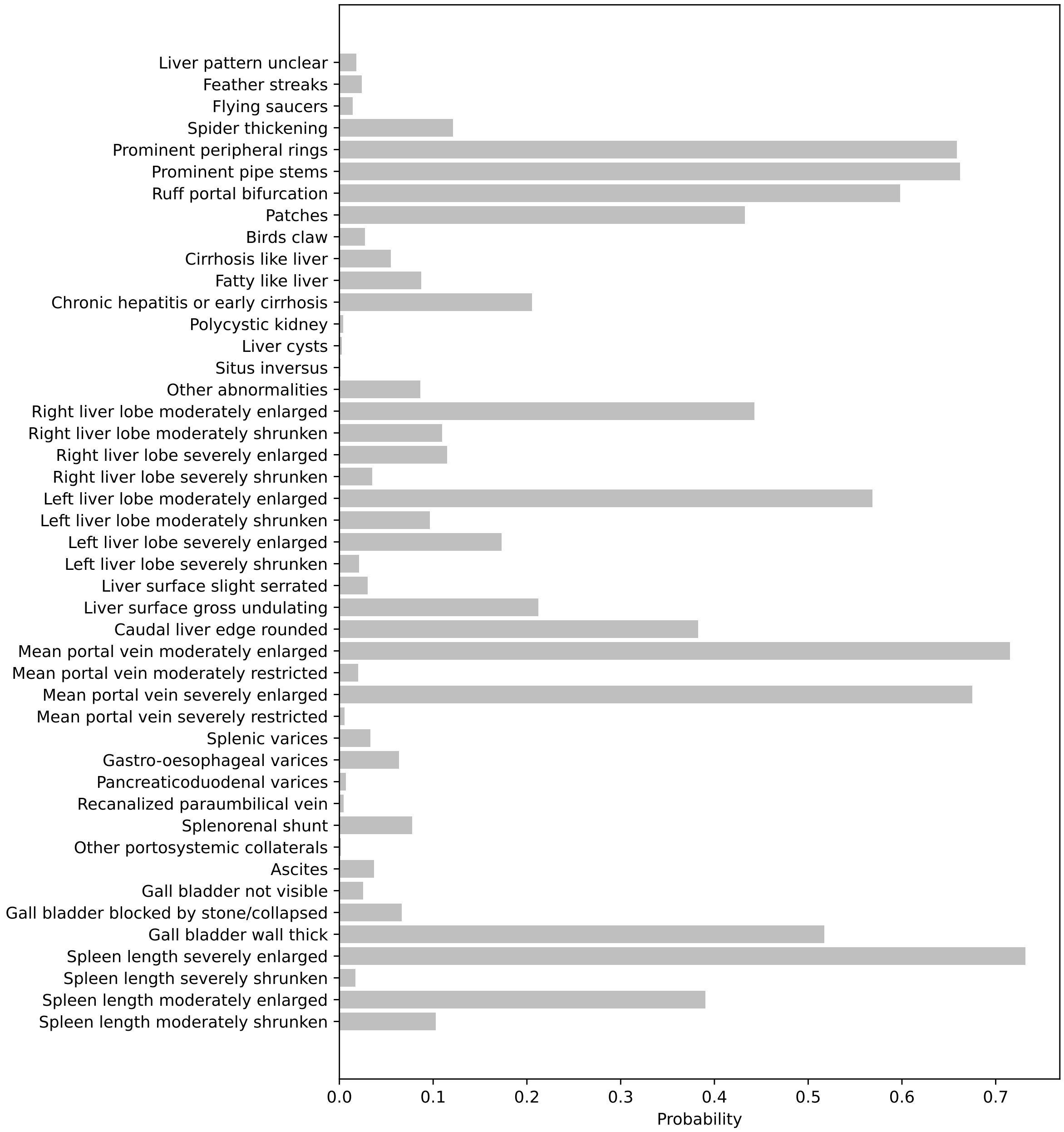

Figure S23: Predicted probabilities of example participants likely to exhibit high expected multimorbidity counts. Example participants were created by assigning the 99th percentiles of the covariate values if the covariate exhibited a significant positive relationship with overall multimorbidity, the 1st percentiles if exhibited a negative significant relationship, and 50th percentile if relationship was not significant. Pakwach was assigned as the district as both were significant but participants can only belong to one district. The expected number of conditions (total probability) was 7.86.

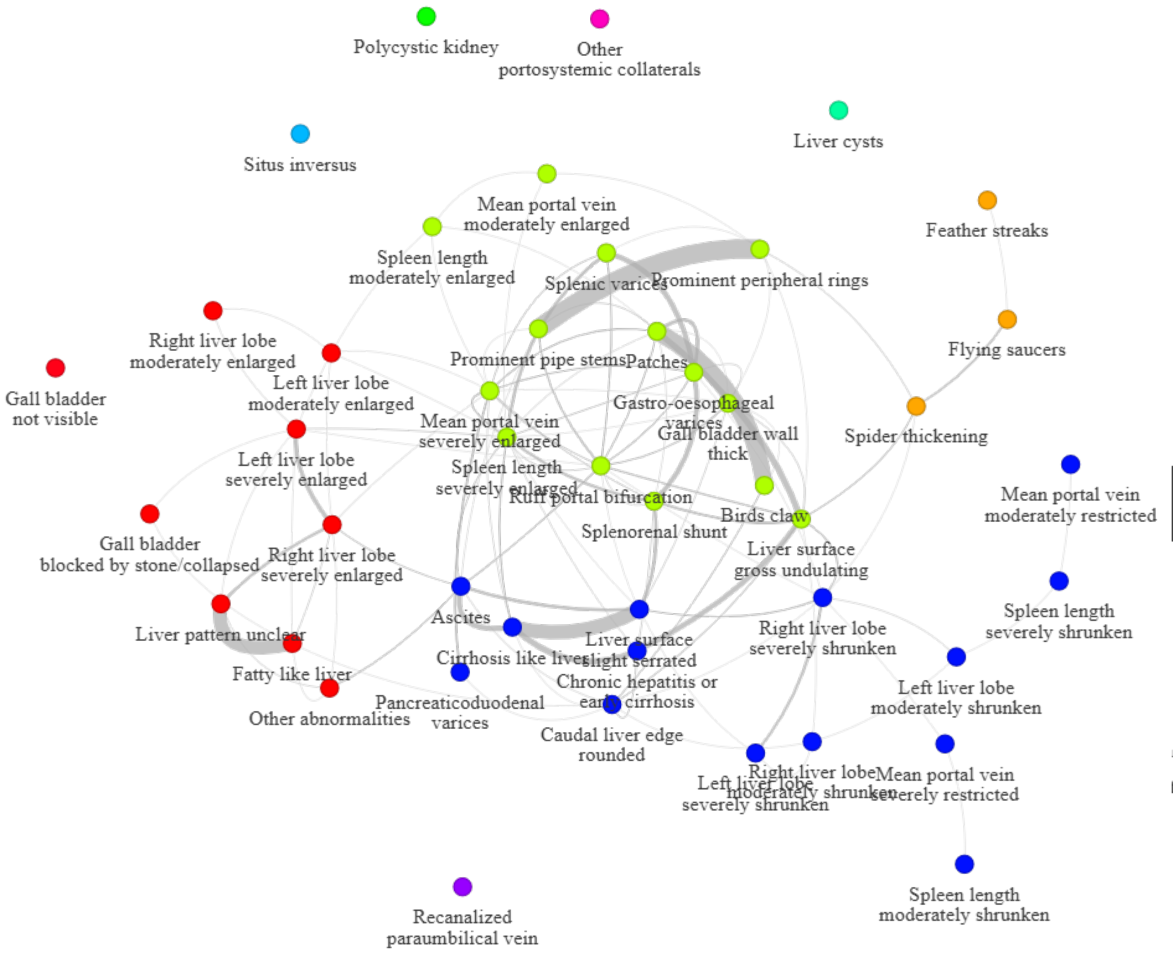

Figure S24: Community detection on the multimorbidity graph using Louvain algorithm. Clusters modularity score: 0.336.

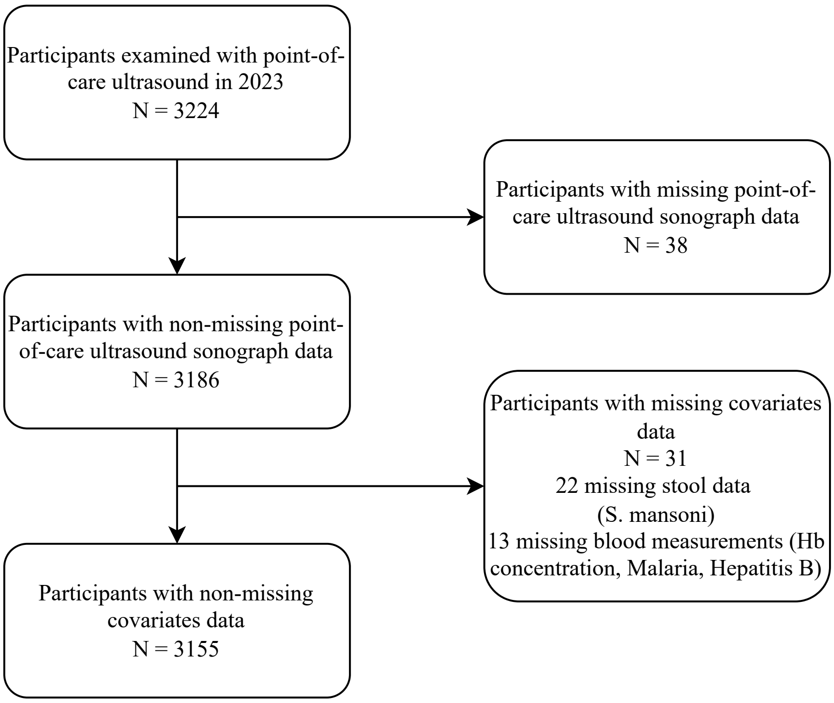

Figure S25: Participants flow diagram.

Condition	None	Mild	Moderate	Severe	χ^2	p_χ
Patches	48.89	13.33	26.67	11.11	6.81	0.08
Patches (absent)	61.90	13.79	21.32	2.99		
Chronic hepatitis or early cirrhosis	38.46	23.08	30.77	7.69	11.86	0.01
Chronic hepatitis or early cirrhosis (absent)	62.20	13.59	21.20	3.01		
Left liver lobe moderately enlarged	55.69	16.11	22.04	6.16	2.08	0.56
Left liver lobe moderately enlarged (absent)	62.64	13.43	21.30	2.63		
Left liver lobe severely enlarged	51.19	14.29	25.00	9.52	4.83	0.18
Left liver lobe severely enlarged (absent)	62.00	13.77	21.30	2.93		
Caudal liver edge rounded	45.26	18.25	28.47	8.03	7.69	0.05
Caudal liver edge rounded (absent)	63.28	13.36	20.72	2.64		
Mean portal vein severely enlarged	53.61	15.06	21.08	10.24	5.08	0.17
Mean portal vein severely enlarged (absent)	62.16	13.72	21.41	2.71		
Gastro-oesophageal varices	27.27	0.00	45.45	27.27	55.38	<0.01
Gastro-oesophageal varices (absent)	61.83	13.84	21.31	3.02		
Splenorenal shunt	15.38	23.08	38.46	23.08	50.68	<0.01
Splenorenal shunt (absent)	61.90	13.75	21.32	3.02		
Ascites	25.00	0.00	33.33	41.67	65.68	<0.01
Ascites (absent)	61.85	13.84	21.35	2.96		
Spleen length severely enlarged	35.56	18.89	36.11	9.44	16.7	<0.01
Spleen length severely enlarged (absent)	63.29	13.48	20.50	2.72		
Spleen length moderately enlarged	49.74	16.41	28.12	5.73	4.2	0.24
Spleen length moderately enlarged (absent)	63.37	13.42	20.46	2.74		

Table S1: Anaemia categories for conditions significant with Hb concentration. Conditions were taken from Table 3. Anaemia category percentages were calculated for each condition over the positive and negative participants separately. Conditions followed by (absent) refer to participants that did not have the condition. χ^2 test is calculated between the percentages of the positive and negative participants of that condition. Results are computed over the full dataset.

Covariate	GOV Median OR (95% CI)	GOV q	PPF Mean OR	PPF q	Overall Mean OR	Overall q
S. mansoni log(EPG+1)	0.94 (0.74, 1.04)	0.78	0.97	0.50	0.99	0.11
Malaria	0.92 (0.52, 1.30)	1.00	0.87	0.50	0.95	<0.01
HBV	1.40 (0.81, 3.72)	1.00	1.21	0.78	1.06	1.00
HIV	2.52 (0.97, 10.77)	0.61	1.77	0.04	1.27	1.00
log(Hb concentration)	0.07 (0.01, 0.37)	0.01	0.66	0.45	0.55	<0.01
Age	1.04 (1.02, 1.08)	0.01	1.04	<0.01	1.02	<0.01
Gender - female	0.67 (0.26, 1.04)	0.61	0.62	<0.01	0.89	<0.01
Majority tribe	1.08 (0.82, 1.56)	1.00	1.10	1.00	1.01	1.00
Majority religion	1.00 (0.79, 1.11)	1.00	1.00	1.00	1.00	1.00
Years in education	1.01 (0.96, 1.08)	1.00	1.02	0.21	1.01	1.00
Farmer	1.17 (0.78, 1.88)	1.00	0.97	1.00	0.99	1.00
Fisherman	1.79 (0.91, 4.42)	0.78	2.00	<0.01	1.23	<0.01
Fishmonger	1.27 (0.63, 3.73)	1.00	1.58	0.12	1.09	1.00
Home quality score	1.00 (0.96, 1.06)	1.00	0.99	0.23	1.00	1.00
Household social status	1.00 (0.76, 1.29)	1.00	1.02	1.00	1.00	1.00
Number of individuals in HH	1.06 (0.96, 1.22)	1.00	1.09	<0.01	1.02	1.00
Years HH lived in village	1.00 (0.99, 1.00)	1.00	1.00	1.00	1.00	1.00
Home owned	0.95 (0.50, 1.50)	1.00	1.05	1.00	1.02	1.00
Number of rooms	0.99 (0.90, 1.05)	1.00	1.00	1.00	1.00	1.00
Current alcohol use	1.01 (0.80, 1.38)	1.00	1.05	1.00	1.00	1.00
Improved drinking water source	1.11 (0.78, 1.73)	1.00	0.95	0.01	1.03	0.19
Number of water activities	1.00 (0.94, 1.07)	1.00	1.00	1.00	1.00	1.00
Year of recruitment - 2023	1.00 (0.78, 1.20)	1.00	0.99	1.00	1.00	1.00
Min. dist. (km) to water site	0.94 (0.61, 1.30)	1.00	0.86	0.20	1.00	0.07
Min. dist. (km) to health centre	1.01 (0.98, 1.06)	1.00	1.01	0.59	1.00	1.00
Buliisa	1.03 (0.57, 2.11)	1.00	1.31	<0.01	1.08	<0.01
Pakwach	1.43 (0.74, 2.82)	1.00	1.99	<0.01	1.28	<0.01

Table S2: Covariates significance on full population.
GOV: gastro-oesophageal varices, PPF: periportal fibrosis, and overall: all 45 conditions. Significance is determined by q-values. For gastro-oesophageal varices, significant covariates are also listed in Table 3, the 95% credible interval is based on the highest posterior density. Significance on periportal fibrosis and overall are computed for grouped outcomes, multiple distributions are combined and potentially multi-modal, therefore the mean are reported instead of medians and credible intervals are not provided as they are not meaningful. Periportal fibrosis combines the densities of the five liver patterns C - F, overall combines all 45 densities. The scales of the odds ratios represented the average per unit rate of each covariate. Results are computed over the full dataset.

Condition	Covariate	Median OR (95% CI)
Feather streaks	Pakwach	1.43 (0.98, 2.05)
Prominent peripheral rings	Age	1.04 (1.03, 1.05)
Prominent peripheral rings	Gender - female	0.61 (0.48, 0.78)
Prominent peripheral rings	Buliisa	1.48 (1.06, 2.05)
Prominent peripheral rings	Pakwach	2.21 (1.55, 3.03)
Prominent pipe stems	Gender - female	0.64 (0.50, 0.77)
Prominent pipe stems	Pakwach	2.06 (1.52, 2.74)
Ruff portal bifurcation	Age	1.08 (1.07, 1.09)
Ruff portal bifurcation	Gender - female	0.53 (0.35, 0.80)
Patches	log(Hb concentration)	0.20 (0.08, 0.58)
Patches	Gender - female	0.60 (0.34, 1.00)
Chronic hepatitis or early cirrhosis	log(Hb concentration)	0.15 (0.06, 0.38)
Chronic hepatitis or early cirrhosis	Pakwach	2.59 (1.30, 5.15)
Left liver lobe moderately enlarged	Buliisa	1.84 (1.30, 2.77)
Left liver lobe moderately enlarged	Pakwach	1.95 (1.40, 2.88)
Left liver lobe severely enlarged	Age	1.05 (1.03, 1.06)
Liver surface gross undulating	log(Hb concentration)	0.21 (0.06, 0.51)
Liver surface gross undulating	Gender - female	0.64 (0.31, 0.95)
Liver surface gross undulating	Pakwach	3.94 (1.57, 11.66)
Caudal liver edge rounded	log(Hb concentration)	0.25 (0.15, 0.47)
Caudal liver edge rounded	Buliisa	1.94 (1.34, 3.01)
Mean portal vein severely enlarged	log(Hb concentration)	0.17 (0.09, 0.34)
Mean portal vein severely enlarged	Age	1.06 (1.05, 1.08)
Mean portal vein severely enlarged	Pakwach	1.94 (1.35, 3.26)
Gastro-oesophageal varices	log(Hb concentration)	0.07 (0.01, 0.37)
Splenorenal shunt	log(Hb concentration)	0.08 (0.02, 0.41)
Ascites	log(Hb concentration)	0.03 (0.01, 0.17)
Gall bladder not visible	Buliisa	4.59 (1.46, 11.80)
Gall bladder wall thick	log(Hb concentration)	0.54 (0.31, 0.86)
Gall bladder wall thick	Gender - female	0.70 (0.52, 0.90)
Gall bladder wall thick	Pakwach	1.52 (1.12, 2.22)
Spleen length severely enlarged	log(Hb concentration)	0.07 (0.03, 0.13)
Spleen length severely enlarged	Pakwach	2.63 (1.51, 4.16)
Spleen length severely shrunken	Age	1.03 (1.01, 1.05)
Spleen length moderately enlarged	Age	1.00 (1.00, 1.01)
Spleen length moderately enlarged	Pakwach	1.78 (1.26, 2.45)
Spleen length moderately shrunken	Age	1.00 (0.99, 1.01)
Spleen length moderately shrunken	Pakwach	0.54 (0.39, 0.80)

Table S3: List of significant relationships found between all conditions and covariates within the adults.
Adults are defined as 18 years or older in the year of study. Outcome conditions and their significant covariates are listed in the first two columns, significance is calculated to 5% based on corrected q-values. Results are computed over the full dataset.

Condition	Covariate	Median OR (95% CI)
Chronic hepatitis or early cirrhosis	log(Hb concentration)	0.05 (0.02, 0.46)
Right liver lobe moderately enlarged	log(Hb concentration)	0.26 (0.20, 0.62)
Left liver lobe moderately enlarged	log(Hb concentration)	0.16 (0.11, 0.44)
Left liver lobe severely enlarged	log(Hb concentration)	0.05 (0.02, 0.38)
Caudal liver edge rounded	log(Hb concentration)	0.16 (0.09, 0.43)
Caudal liver edge rounded	Min. dist. (km) to water site	3.44 (1.80, 4.07)
Mean portal vein moderately enlarged	log(Hb concentration)	0.23 (0.17, 0.66)
Gall bladder not visible	Buliisa	2.77 (0.90, 5.45)
Gall bladder blocked by stone/collapsed	Gender - female	0.63 (0.25, 0.95)
Spleen length severely enlarged	Age	1.18 (1.05, 1.29)
Spleen length severely shrunken	log(Hb concentration)	0.16 (0.02, 0.59)
Spleen length moderately enlarged	Malaria	3.26 (2.83, 6.16)
Spleen length moderately enlarged	log(Hb concentration)	0.15 (0.09, 0.34)
Spleen length moderately shrunken	Malaria	0.16 (0.14, 0.25)
Spleen length moderately shrunken	Age	0.83 (0.80, 0.91)

Table S4: List of significant relationships found between all conditions and covariates within children.
Children are defined as 5-17 years old in the year of study. Outcome conditions and their significant covariates are listed in the first two columns, significance is calculated to 5% based on corrected q-values. Results are computed over the full dataset.

Covariate	GOV Median OR (95% CI)	GOV q	PPF Mean OR	PPF q	Overall Mean OR	Overall q
S. mansoni log(EPG+1)	0.93 (0.72, 1.05)	0.67	0.97	0.15	0.97	1.00
Malaria	0.91 (0.49, 1.34)	1.00	0.86	1.00	0.86	1.00
HBV	1.31 (0.85, 2.89)	1.00	1.16	1.00	1.16	1.00
HIV	2.12 (0.98, 6.92)	0.80	1.59	0.03	1.59	1.00
log(Hb concentration)	0.07 (0.01, 0.37)	<0.01	0.66	0.01	0.66	<0.01
Age	1.06 (1.03, 1.10)	1.00	1.05	<0.01	1.05	<0.01
Gender - female	0.66 (0.25, 1.04)	0.09	0.61	<0.01	0.61	<0.01
Majority tribe	1.08 (0.82, 1.56)	1.00	1.10	1.00	1.10	1.00
Majority religion	1.00 (0.79, 1.10)	1.00	1.00	1.00	1.00	1.00
Years in education	1.01 (0.96, 1.07)	1.00	1.02	1.00	1.02	1.00
Farmer	1.13 (0.82, 1.66)	1.00	0.98	0.33	0.98	1.00
Fisherman	1.54 (0.93, 3.03)	1.00	1.68	0.07	1.68	1.00
Fishmonger	1.19 (0.71, 2.60)	1.00	1.39	0.88	1.39	1.00
Home quality score	1.00 (0.96, 1.06)	1.00	0.99	0.23	0.99	1.00
Household social status	1.00 (0.76, 1.29)	1.00	1.02	1.00	1.02	1.00
Number of individuals in HH	1.06 (0.96, 1.22)	1.00	1.09	0.03	1.09	1.00
Years HH lived in village	1.00 (0.99, 1.00)	1.00	1.00	1.00	1.00	1.00
Home owned	0.95 (0.51, 1.50)	1.00	1.05	1.00	1.05	1.00
Number of rooms	0.99 (0.90, 1.05)	1.00	1.00	1.00	1.00	1.00
Current alcohol use	1.01 (0.84, 1.28)	1.00	1.04	1.00	1.04	1.00
Improved drinking water source	1.11 (0.78, 1.73)	1.00	0.95	0.18	0.95	1.00
Number of water activities	1.00 (0.94, 1.07)	1.00	1.00	1.00	1.00	1.00
Year of recruitment - 2023	1.00 (0.78, 1.20)	1.00	0.99	1.00	0.99	1.00
Min. dist. (km) to water site	0.94 (0.61, 1.30)	1.00	0.85	0.08	0.85	1.00
Min. dist. (km) to health centre	1.01 (0.98, 1.06)	1.00	1.01	1.00	1.01	1.00
Buliisa	1.03 (0.56, 2.11)	1.00	1.31	0.01	1.31	<0.01
Pakwach	1.43 (0.74, 2.83)	1.00	1.99	<0.01	1.99	<0.01

Table S5: Covariates significance on adults.
GOV: gastro-oesophageal varices, PPF: periportal fibrosis, and overall: all 45 conditions. Significance is determined by q-values. For gastro-oesophageal varices, significant covariates are also listed in Table S3, the 95% credible interval is based on the highest posterior density. Significance on periportal fibrosis and overall are computed for grouped outcomes, multiple distributions are combined and potentially multi-modal, therefore the mean are reported instead of medians and credible intervals are not provided as they are not meaningful. Periportal fibrosis combines the densities of the five liver patterns C - F, overall combines all 45 densities. The scales of the odds ratios represented the average per unit rate of each covariate. Results are computed over the full dataset.

Covariate	GOV Median OR (95% CI)	GOV q	PPF Mean OR	PPF q	Overall Mean OR	Overall q
S. mansoni log(EPG+1)	1.04 (0.93, 1.11)	1.00	1.00	1.00	1.00	1.00
Malaria	1.11 (0.43, 1.80)	1.00	0.97	1.00	1.02	<0.01
HBV	0.72 (0.38, 1.91)	1.00	1.03	1.00	0.98	1.00
HIV	1.23 (0.57, 2.04)	1.00	1.01	1.00	1.01	1.00
log(Hb concentration)	0.39 (0.07, 3.76)	1.00	1.40	1.00	0.57	<0.01
Age	1.03 (0.93, 1.20)	1.00	1.04	1.00	1.02	<0.01
Gender - female	0.93 (0.68, 1.57)	1.00	1.04	1.00	0.96	1.00
Majority tribe	1.00 (0.81, 1.47)	1.00	1.00	1.00	1.00	1.00
Majority religion	0.88 (0.82, 1.33)	1.00	0.99	1.00	1.00	1.00
Years in education	0.97 (0.92, 1.07)	1.00	1.00	1.00	1.00	1.00
Farmer	0.94 (0.37, 3.09)	1.00	1.03	1.00	0.99	1.00
Fisherman	0.06 (0.01, 2.19)	1.00	0.51	1.00	0.88	1.00
Home quality score	0.98 (0.97, 1.03)	1.00	1.00	1.00	1.00	1.00
Household social status	1.22 (0.68, 1.35)	1.00	1.02	1.00	1.01	1.00
Number of individuals in HH	1.05 (0.95, 1.11)	1.00	1.00	1.00	1.00	1.00
Years HH lived in village	1.00 (1.00, 1.00)	1.00	1.00	1.00	1.00	1.00
Home owned	1.06 (0.63, 1.40)	1.00	1.02	1.00	1.02	1.00
Number of rooms	1.00 (0.94, 1.05)	1.00	1.00	1.00	1.00	1.00
Current alcohol use	1.24 (0.35, 2.40)	1.00	0.72	1.00	0.95	1.00
Improved drinking water source	1.23 (0.79, 1.36)	1.00	0.95	1.00	0.99	1.00
Number of water activities	1.00 (0.94, 1.04)	1.00	1.00	1.00	1.00	1.00
Year of recruitment - 2023	1.21 (0.70, 2.12)	1.00	1.01	1.00	1.01	1.00
Min. dist. (km) to water site	1.36 (0.50, 1.49)	1.00	0.99	1.00	1.02	0.15
Min. dist. (km) to health centre	1.00 (0.97, 1.05)	1.00	1.02	1.00	1.00	1.00
Buliisa	1.69 (0.74, 2.35)	1.00	1.02	1.00	1.01	1.00
Pakwach	1.20 (0.79, 1.44)	1.00	1.07	1.00	1.03	1.00

Table S6: Covariates significance on children.
GOV: gastro-oesophageal varices, PPF: periportal fibrosis, and overall: all 45 conditions. Significance is determined by q-values. For gastro-oesophageal varices, the 95% credible interval is based on the highest posterior density. Significance on periportal fibrosis and overall are computed for grouped outcomes, multiple distributions are combined and potentially multi-modal, therefore the mean are reported instead of medians and credible intervals are not provided as they are not meaningful. Periportal fibrosis combines the densities of the five liver patterns C - F, overall combines all 45 densities. The scales of the odds ratios represented the average per unit rate of each covariate. Results are computed over the full dataset.

Model	F1 (Macro)	Prec (Macro)	Rec (Macro)	NPV (90%)	BA
LR (AIC)	0.594 ± 0.003	0.578 ± 0.004	0.633 ± 0.010	0.988 ± 0.001	0.662 ± 0.013
XGBoost	0.593 ± 0.001	0.586 ± 0.004	0.637 ± 0.010	0.988 ± 0.001	0.676 ± 0.017
MO-RF	0.590 ± 0.003	0.578 ± 0.006	0.612 ± 0.001	0.988 ± 0.001	0.672 ± 0.015
ST-NN	0.595 ± 0.004	0.581 ± 0.006	0.610 ± 0.015	0.988 ± 0.001	0.663 ± 0.017
MT-NN	0.598 ± 0.003	0.583 ± 0.004	0.630 ± 0.010	0.988 ± 0.001	0.676 ± 0.013
BMO-LR	0.598 ± 0.002	0.580 ± 0.004	0.624 ± 0.008	0.986 ± 0.001	0.656 ± 0.008
BMT-G	0.598 ± 0.002	0.581 ± 0.003	0.638 ± 0.014	0.988 ± 0.001	0.678 ± 0.011
BMT-CL	0.601 ± 0.003	0.585 ± 0.005	0.634 ± 0.016	0.987 ± 0.001	0.671 ± 0.007
BMT-G&CL	0.601 ± 0.003	0.585 ± 0.005	0.636 ± 0.014	0.987 ± 0.001	0.675 ± 0.011

Table S7: Model performance comparison.
Model abbreviations: LR (AIC): Logistic regression AIC variable selection, MO-RF: Multi-output random forest, ST-NN: Single task neural network, MT-NN: Multitask neural network, BMO-LR: Bayesian multi-output logistic regression, BMT-G: Bayesian multitask with graph, BMT-CL: Bayesian multitask with covariate learning, BMT-G&CL: Bayesian multitask with graph and covariate learning. Metrics: For F1, precision (Prec), and recall (Rec), predicted probabilities are converted to binary using a first cut-off based on the highest F1 score, the three metrics are reported using the "macro" option in sklearn. Negative predicted value (NPV) is computed using a second cut-off found based on to 90% sensitivity. All metrics reported were evaluated on 10 randomly sampled test sets of 50% of the data using models trained on the other 50%.

Metric	Test on Pakwach	Test on Buliisa	Test on Mayuge
AUC	0.668 ± 0.012	0.646 ± 0.022	0.661 ± 0.018
AUC-PR	0.108 ± 0.009	0.110 ± 0.017	0.138 ± 0.038
F1 (Bin)	0.202 ± 0.007	0.190 ± 0.008	0.186 ± 0.006
Prec (Bin)	0.144 ± 0.010	0.133 ± 0.009	0.110 ± 0.005
Rec (Bin)	0.350 ± 0.056	0.339 ± 0.036	0.616 ± 0.055
F1 (Macro)	0.568 ± 0.003	0.564 ± 0.005	0.553 ± 0.005
Prec (Macro)	0.559 ± 0.004	0.556 ± 0.005	0.556 ± 0.004
Rec (Macro)	0.587 ± 0.012	0.583 ± 0.012	0.587 ± 0.015
PPV	0.089 ± 0.003	0.084 ± 0.003	0.090 ± 0.004
NPV	0.982 ± 0.001	0.985 ± 0.001	0.989 ± 0.001

Table S8: Performance of Bayesian multitask learning with districts-based leave-one-out data splitting.
Data split was based on districts, the Bayesian multitask model was trained on participants from two districts, and tested bootstrapped samples of the remaining. The district overall prevalences are Pakwach: 0.052, Buliisa: 0.045, Mayuge: 0.038.

Node 1	Node 2	Probability
Mean portal vein severely restricted	Spleen length moderately shrunken	0.294
Prominent peripheral rings	Prominent pipe stems	0.278
Mean portal vein moderately restricted	Spleen length severely shrunken	0.276
Liver pattern unclear	Fatty like liver	0.253
Feather streaks	Flying saucers	0.216
Flying saucers	Spider thickening	0.206
Patches	Birds claw	0.175
Cirrhosis like liver	Liver surface slight serrated	0.150
Right liver lobe moderately enlarged	Left liver lobe moderately enlarged	0.149
Right liver lobe severely enlarged	Left liver lobe severely enlarged	0.142
Left liver lobe moderately shrunken	Spleen length severely shrunken	0.139
Right liver lobe severely shrunken	Left liver lobe severely shrunken	0.132
Splenic varices	Gastro-oesophageal varices	0.119
Mean portal vein moderately enlarged	Spleen length moderately enlarged	0.117
Right liver lobe moderately shrunken	Left liver lobe moderately shrunken	0.100
Right liver lobe moderately enlarged	Left liver lobe severely enlarged	0.098
Mean portal vein severely enlarged	Pancreaticoduodenal varices	0.096
Chronic hepatitis or early cirrhosis	Liver surface gross undulating	0.096
Liver pattern unclear	Right liver lobe severely enlarged	0.089
Splenic varices	Spleen length severely enlarged	0.085

Table S9: Highest probability pairs in the graph convolutional matrix. Results are computed over the full dataset.
