# Supplementary material for: Bayesian machine learning enables discovery of risk factors for hepatosplenic multimorbidity related to schistosomiasis
Source: Nat Commun. 2026 Mar 3;17:3377. doi: 10.1038/s41467-026-69528-4 (PMC13066555; doi:10.1038/s41467-026-69528-4)
Supplement: Supplementary file 3 — Description of Additional Supplementary Files [file 41467_2026_69528_MOESM3_ESM.pdf]

Description of Additional Supplementary Files

File Name: Supplementary Code 1

Description: Implementation of the Bayesian multitask model introduced in the paper.

The model was implemented using probabilistic programming library Pyro, based on PyTorch, in addition to standard libraries in Python. The provided node and edge lists corresponded to the graph used in the paper, synthetic input covariates and outcomes were attached to allow the code to run. To adapt the code to your own problems, users can import their own set of inputs and outcome data. If a graph exists, the user can pass in the node and edge lists, otherwise use `model_id` which runs the Bayesian multitask model without the graph.
