# Supplementary material for: Bayesian machine learning enables discovery of risk factors for hepatosplenic multimorbidity related to schistosomiasis
Source: Nat Commun. 2026 Mar 3;17:3377. doi: 10.1038/s41467-026-69528-4 (PMC13066555; doi:10.1038/s41467-026-69528-4)
Supplement: Supplementary file 5 — Reporting Summary [file 41467_2026_69528_MOESM5_ESM.pdf]

nature portfolio | reporting summary

nature portfolio

Corresponding author(s): Goylette F. Chami

Last updated by author(s): Dec 12, 2025

Reporting Summary

Nature Portfolio wishes to improve the reproducibility of the work that we publish. This form provides structure for consistency and transparency in reporting. For further information on Nature Portfolio policies, see our Editorial Policies and the Editorial Policy Checklist.

Statistics

For all statistical analyses, confirm that the following items are present in the figure legend, table legend, main text, or Methods section.

n/a	Confirmed	
☐	☒	The exact sample size (*n*) for each experimental group/condition, given as a discrete number and unit of measurement
☒	☐	A statement on whether measurements were taken from distinct samples or whether the same sample was measured repeatedly
☐	☒	The statistical test(s) used AND whether they are one- or two-sided<br>*Only common tests should be described solely by name; describe more complex techniques in the Methods section.*
☐	☒	A description of all covariates tested
☐	☒	A description of any assumptions or corrections, such as tests of normality and adjustment for multiple comparisons
☐	☒	A full description of the statistical parameters including central tendency (e.g. means) or other basic estimates (e.g. regression coefficient) AND variation (e.g. standard deviation) or associated estimates of uncertainty (e.g. confidence intervals)
☐	☒	For null hypothesis testing, the test statistic (e.g. *F*, *t*, *r*) with confidence intervals, effect sizes, degrees of freedom and *P* value noted<br>*Give P values as exact values whenever suitable.*
☐	☒	For Bayesian analysis, information on the choice of priors and Markov chain Monte Carlo settings
☐	☒	For hierarchical and complex designs, identification of the appropriate level for tests and full reporting of outcomes
☐	☒	Estimates of effect sizes (e.g. Cohen's *d*, Pearson's *r*), indicating how they were calculated

Our web collection on statistics for biologists contains articles on many of the points above.

Software and code

Policy information about availability of computer code

Data collection	Open Data Kit (ODK) was used for data collection (ODK tablet app version v2021.3.1, ODK XLSForm Offline v1.9.0)
Data analysis	Python 3.10 was used for data analysis

For manuscripts utilizing custom algorithms or software that are central to the research but not yet described in published literature, software must be made available to editors and reviewers. We strongly encourage code deposition in a community repository (e.g. GitHub). See the Nature Portfolio guidelines for submitting code & software for further information.

Data

Policy information about availability of data

All manuscripts must include a data availability statement. This statement should provide the following information, where applicable:

- Accession codes, unique identifiers, or web links for publicly available datasets
- A description of any restrictions on data availability
- For clinical datasets or third party data, please ensure that the statement adheres to our policy

Data is not publicly available due to data protection and ethics restrictions related to the ongoing nature of the SchistoTrack Cohort and easily identifiable nature of the participants. Synthetically generated data is provided based on random sampling of the covariates and conditions to allow the running of the code.

Research involving human participants, their data, or biological material

Policy information about studies with human participants or human data. See also policy information about sex, gender (identity/presentation), and sexual orientation and race, ethnicity and racism.

Reporting on sex and gender	We exclusively use the term gender, following the definitions laid out in the 'Sex and Gender Equity in Research – SAGER – guidelines' because in this study, information was elicited via self-reports and we sought to understand how gender (shaped by social and cultural norms) influences water contact behaviour. We discuss the implications of gender on the outcomes in the Discussion section. We have noted in the reporting guidelines that gender was self-reported and that gender was not disaggregated differently for the experiments in this study. Gender, age, and number of participants are all summarized in Table 2.
Reporting on race, ethnicity, or other socially relevant groupings	We recorded self-reported data on tribe (Alur, Banyoro, Bagungu, Musiki, Musoga, Jaluo, Mulamongi, Musamya, Muganda, Mudama, Muteso, Other, Mugwere, Lugbara, Munyole, Kakwa, Mukenye, Mugisu, Munyarwanda, Munyankole, Baganda, Mukiga, Madi, Balalo). We also recorded self-reported data on religion (Christian, Muslim, Born-again christian, no religion). However, in our analyses these variables were not selected for inclusion in the main models. We do not isolate individual tribes or religions in the analysis and instead only report on majority tribe and majority religion as per village.
Population characteristics	See behavioral & social study design section below
Recruitment	We randomly sampled a total of 1952 households from 52 villages across 3 districts. All households with at least one child and one adult residing in the village for at least six months of the year were eligible.
Ethics oversight	Data collection and use were reviewed and approved by Oxford Tropical Research Ethics Committee (OxTREC 509-21), Vector Control Division Research Ethics Committee of the Uganda Ministry of Health (VCDREC146), and Uganda National Council of Science and Technology (UNCST HS 1664ES). Written informed consent was obtained for adult participants, with adults consenting on behalf of the children after receiving their informed assent. Ethics approval was obtained from all local ethics committees. Participants were compensated in-kind for their time with a large (1kg) bar of soap.

Note that full information on the approval of the study protocol must also be provided in the manuscript.

Field-specific reporting

Please select the one below that is the best fit for your research. If you are not sure, read the appropriate sections before making your selection.

☐ Life sciences ☐ Behavioural & social sciences ☒ Ecological, evolutionary & environmental sciences

For a reference copy of the document with all sections, see nature.com/documents/nr-reporting-summary-flat.pdf

Ecological, evolutionary & environmental sciences study design

All studies must disclose on these points even when the disclosure is negative.

Study description	Cross-sectional study of 3155 individuals aged 5-91 years in Eastern and Western Uganda. We collected socio-demographics, biomedical information (including schistosome infection status), environmental data, locational data.
Research sample	Population representative sample of 3155 participants across 52 villages aged 5-91, 1457 females and 1698 males
Sampling strategy	We randomly sampled a total of 1952 households from 52 villages across 3 districts in Uganda, from village registers or MDA records. All households with at least one child and one adult residing in the village for at least six months of the year were eligible. Based on the schistosomiasis prevalence recorded in a previous study in one of the study areas (Maygue) conducted by GFC and NBK, this study was designed to detect a minimum effect size of 8% with an unevenly exposed population and a household design effect of 1.136 at 97.5% power.
Data collection	Household data collection was done by trained surveyors (either from Kampala or locally recruited) using tables and ODK software. Household-level information was collected prior to clinical data collection and participants were unaware of their infection status. All clinical assessments were conducted by technicians/nurses from the district or Kampala in mobile in-the-field labs.
Timing and spatial scale	Data collection took place in January to February 2023 in Pakwach, Buliisa and Mayuge.
Data exclusions	A flow chart detailing any data exclusions is provided in Figure S25. We initially recruited 3224 participants for clinical assessments but due to missing data and non-participation, the number of participants used for analysis was 3155.
Reproducibility	This was not an experimental study. However, to enable reproducibility of the data collection and analyses, detailed methods and step-by-step procedures are provided in the manuscript as well as the necessary implementation and synthetically generated data to allow the running of the code.
Randomization	N/A as this was an observational study.
Blinding	N/A. However, participants were not aware of their infection status when reporting in the household survey and the researchers used a data-driven approach to the variable selection that was not influenced by purposeful selection of variables.

Did the study involve field work? ☒ Yes ☐ No

Field work, collection and transport

Field conditions	This study was conducted during the dry season after flooding had occurred in Uganda and started to recede in early 2023.
Location	The location was in 52 villages across three districts including Pakwach, Buliisa, and Uganda. There were no experimental ecological studies done that required exact sites to be reported.
Access & import/export	N/A - there was no biobanking in this study.
Disturbance	A full data protection impact assessment was completed, and participants were compensated for any time lost spent in the study. There was no ecological disturbance as a result of this study.

Reporting for specific materials, systems and methods

We require information from authors about some types of materials, experimental systems and methods used in many studies. Here, indicate whether each material, system or method listed is relevant to your study. If you are not sure if a list item applies to your research, read the appropriate section before selecting a response.

Materials & experimental systems

n/a	Involved in the study
☒	☐ Antibodies
☒	☐ Eukaryotic cell lines
☒	☐ Palaeontology and archaeology
☒	☐ Animals and other organisms
☐	☒ Clinical data
☒	☐ Dual use research of concern
☒	☐ Plants

Methods

n/a	Involved in the study
☒	☐ ChIP-seq
☒	☐ Flow cytometry
☒	☐ MRI-based neuroimaging

Clinical data

Policy information about clinical studies
All manuscripts should comply with the ICMJE guidelines for publication of clinical research and a completed CONSORT checklist must be included with all submissions.

Clinical trial registration	Not applicable as this was an observational study.
Study protocol	Not applicable as this was an observational study.
Data collection	Data collection took place in January to February 2022 in Pakwach, Buliisa and Mayuge.
Outcomes	Outcomes were obtained from point-of-care ultrasound and consisted of 45 hepatosplenic health conditions.

Plants

Seed stocks	Not applicable
Novel plant genotypes	Not applicable
Authentication	Not applicable
